# Plasma polyphenols associated with lower high-sensitivity C-reactive protein concentrations: a cross-sectional study within the European Prospective Investigation into Cancer and Nutrition (EPIC) cohort

**DOI:** 10.1017/S0007114519002538

**Published:** 2020-01-28

**Authors:** Laura M. Harms, Augustin Scalbert, Raul Zamora-Ros, Sabina Rinaldi, Mazda Jenab, Neil Murphy, David Achaintre, Anne Tjønneland, Anja Olsen, Kim Overvad, Francesca Romana Mancini, Yahya Mahamat-Saleh, Marie-Christine Boutron-Ruault, Tilman Kühn, Verena Katzke, Antonia Trichopoulou, Georgia Martimianaki, Anna Karakatsani, Domenico Palli, Salvatore Panico, Sabina Sieri, Rosario Tumino, Carlotta Sacerdote, Bas Bueno-de-Mesquita, Roel C. H. Vermeulen, Elisabete Weiderpass, Therese Haugdahl Nøst, Cristina Lasheras, Miguel Rodríguez-Barranco, José María Huerta, Aurelio Barricarte, Miren Dorronsoro, Johan Hultdin, Julie A. Schmidt, Marc Gunter, Elio Riboli, Krasimira Aleksandrova

**Affiliations:** 1 Nutrition, Immunity and Metabolism Senior Scientist Group, Department of Nutrition and Gerontology, German Institute of Human Nutrition Potsdam-Rehbruecke (DIfE), 14558 Nuthetal, Germany; 2 International Agency for Research on Cancer, World Health Organization, 69008 Lyon, France; 3 Unit of Nutrition and Cancer, Cancer Epidemiology Research Programme, Catalan Institute of Oncology, Bellvitge Biomedical Research Institute (IDIBELL), 08908 Barcelona, Spain; 4 Danish Cancer Society Research Center, 2100 Copenhagen, Denmark; 5 Department of Public Health, University of Copenhagen, 2200 Copenhagen, Denmark; 6 Department of Public Health, Aarhus University, DK-8000 Aarhus C, Denmark; 7 Department of Cardiology, Aalborg University Hospital, 9100 Aalborg, Denmark; 8 CESP, faculté de médecine, université Paris-Sud, 75006 Paris, France; 9 UVSQ, INSERM, Université Paris-Saclay, 94805 Villejuif, France; 10 Division of Cancer Epidemiology, German Cancer Research Center (DKFZ), 69120 Heidelberg, Germany; 11 Hellenic Health Foundation, 11527 Athens, Greece; 12 WHO Collaborating Center for Nutrition and Health, Unit of Nutritional Epidemiology and Nutrition in Public Health, Department of Hygiene, Epidemiology and Medical Statistics, School of Medicine, National and Kapodistrian University of Athens, 15772 Athens, Greece; 13 2nd Pulmonary Medicine Department, School of Medicine, National and Kapodistrian University of Athens, “ATTIKON” University Hospital, 12462 Chaidari, Greece; 14 Molecular and Nutritional Epidemiology Unit, Cancer Research and Prevention Institute– ISPO, 50139 Firenze, Italy; 15 EPIC Centre of Naples, Dipartimento di Medicina Clinica e Chirurgia Federico II University, 80131 Napoli, Italy; 16 Epidemiology and Prevention Unit Fondazione Istituto Nazionale dei Tumori di Milano, 20133 Milano, Italy; 17 Cancer Registry and Histopathology Unit, “Civic–M.P. Arezzo” Hospital, 97100 Ragusa, Italy; 18 Unit of Cancer Epidemiology, Città della Salute e della Scienza University-Hospital and Center for Cancer Prevention (CPO), 10126 Turin, Italy; 19 Department of Epidemiology and Biostatistics, School of Public Health, Imperial College London, London SW7 2AZ, UK; 20 National Institute for Public Health and the Environment (RIVM), 3720 BA Bilthoven, The Netherlands; 21 Department of Gastroenterology and Hepatology, University Medical Centre, 3584 CX Utrecht, The Netherlands; 22 Department of Social and Preventative Medicine, Faculty of Medicine, University of Malaya, 50603 Kuala Lumpur, Malaysia; 23 Division of Environmental Epidemiology, Institute for Risk Assessment Sciences, Utrecht University, 3584 CX Utrecht, The Netherlands; 24 Department of Epidemiology, Julius Center for Health Sciences and Primary Care, University Medical Center Utrecht, 3584 CG Utrecht, The Netherlands; 25 Department of Community Medicine, University of Tromsø, The Arctic University of Norway, 9019 Tromsø, Norway; 26 Department of Functional Biology, Faculty of Medicine, University of Oviedo, 33006 Oviedo, Spain; 27 Andalusian School of Public Health (EASP), Instituto de Investigación Biosanitaria de Granada (ibs.GRANADA), Universidad de Granada, 18011 Granada, Spain; 28 CIBER de Epidemiología y Salud Pública (CIBERESP), 28029 Madrid, Spain; 29 Department of Epidemiology, Murcia Regional Health Council, IMIB-Arrixaca, 30008 Murcia, Spain; 30 Navarra Public Health Institute, 31002 Pamplona, Spain; 31 Navarra Institute for Health Research (IdiSNA), 31008 Pamplona, Spain; 32 Public Health Direction and Biodonostia-Ciberesp, Basque Regional Health Department, 20014 Donostia-San Sebastián, Spain; 33 Umeå University, Medical Biosciences, Clinical Chemistry, 901 87 Umeå, Sweden; 34 Cancer Epidemiology Unit, Nuffield Department of Population Health, University of Oxford, Oxford OX3 7LF, UK; 35 University of Potsdam, Institute of Nutritional Science, 14558 Nuthetal, Germany

**Keywords:** Polyphenols, Plasma measurements, C-reactive protein, Inflammation, Chronic diseases

## Abstract

Experimental studies have reported on the anti-inflammatory properties of polyphenols. However, results from epidemiological investigations have been inconsistent and especially studies using biomarkers for assessment of polyphenol intake have been scant. We aimed to characterise the association between plasma concentrations of thirty-five polyphenol compounds and low-grade systemic inflammation state as measured by high-sensitivity C-reactive protein (hsCRP). A cross-sectional data analysis was performed based on 315 participants in the European Prospective Investigation into Cancer and Nutrition cohort with available measurements of plasma polyphenols and hsCRP. In logistic regression analysis, the OR and 95 % CI of elevated serum hsCRP (>3 mg/l) were calculated within quartiles and per standard deviation higher level of plasma polyphenol concentrations. In a multivariable-adjusted model, the sum of plasma concentrations of all polyphenols measured (per standard deviation) was associated with 29 (95 % CI 50, 1) % lower odds of elevated hsCRP. In the class of flavonoids, daidzein was inversely associated with elevated hsCRP (OR 0·66, 95 % CI 0·46, 0·96). Among phenolic acids, statistically significant associations were observed for 3,5-dihydroxyphenylpropionic acid (OR 0·58, 95 % CI 0·39, 0·86), 3,4-dihydroxyphenylpropionic acid (OR 0·63, 95 % CI 0·46, 0·87), ferulic acid (OR 0·65, 95 % CI 0·44, 0·96) and caffeic acid (OR 0·69, 95 % CI 0·51, 0·93). The odds of elevated hsCRP were significantly reduced for hydroxytyrosol (OR 0·67, 95 % CI 0·48, 0·93). The present study showed that polyphenol biomarkers are associated with lower odds of elevated hsCRP. Whether diet rich in bioactive polyphenol compounds could be an effective strategy to prevent or modulate deleterious health effects of inflammation should be addressed by further well-powered longitudinal studies.

The aetiological role of chronic low-grade inflammation in the development of a plethora of chronic diseases including CVD and cancer has been long recognised^(1,2)^. Targeting inflammation could therefore represent an effective approach for preventing onset of chronic diseases^(3)^. Recent evidence has suggested that inflammatory biomarkers such as high-sensitivity C-reactive protein (hsCRP)^(4)^ could be successfully modulated following consumption of plant-originated foods such as whole grains, fruits, vegetables, nuts and olive oil (Mediterranean-style diet)^(5,6)^. Plant-based foods contain high quantities of polyphenols, a large group of plant secondary metabolites with a growing body of evidence indicating beneficial effects on overall health^(7)^. Studies exploring the link between polyphenols and inflammation *in vitro* or in animal models have suggested antioxidative and anti-inflammatory properties for specific polyphenol compounds^(8)^. However, to what extent these results could be translated to free-living humans remains unclear^(9)^. Several epidemiological studies have evaluated the association between intake of selected dietary polyphenols and inflammatory biomarkers providing inconsistent evidence^(10)^. Interpretation of data from these studies is challenged by measurement inaccuracies and inter-individual variability of self-reported polyphenol intakes. Many ingested polyphenols are absorbed in the gut and eventually transformed by the gut microbiota and/or host tissues into metabolites that have been used as biomarkers of intake^(11)^. Measurements of polyphenols and their metabolites in plasma could provide more reliable estimates of exposure, yet studies employing biomarkers of polyphenol intake have been scant and limited to evaluation of specific polyphenol compounds^(12–16)^.

The aim of the present analysis was to characterise the association between plasma concentrations of thirty-five polyphenol compounds and state of low-grade inflammation as measured by hsCRP taking into account various factors of potential influence in a well-phenotyped cross-sectional sample from the European Prospective Investigation into Cancer and Nutrition (EPIC) cohort.

## Methods

### Study population and collection of blood samples and data

EPIC is a multicentre prospective cohort of 521 330 participants, aged ≥35 years, who were recruited in 1992–2000, predominantly from the general population of ten European countries, including France, Italy, Spain, the UK, The Netherlands, Greece, Germany, Sweden, Denmark and Norway^(17)^.

The flow chart of study population selection for the present analysis is described in online Supplementary Fig. S1. Among all EPIC participants, 387 889 provided blood samples. Among these, 5235 participants who were alive and free of major chronic diseases, that is, cancer, served as healthy controls in previous analyses where concentrations of hsCRP^(18)^ and polyphenols^(19)^ have been measured. Among these, 4061 participants were excluded due to missing hsCRP measurements, leaving a sample of 1174 participants. Of them, further 859 participants were excluded due to lack of available polyphenol measurements, providing a final analytical study sample of 315 participants.

As previously reported, blood samples were collected according to standardised procedures and stored at the International Agency for Research on Cancer (−196°C, liquid N_2_) for all countries except Denmark (−150°C, nitrogen-vapour) and Sweden (−80°C, freezers)^(17)^. Participants completed standardised questionnaires on socio-demographic and lifestyle characteristics and personal history at recruitment, and most participants also had anthropometric measurements and blood samples taken at recruitment before disease onset or diagnosis. Dietary intakes over the previous 12 months were assessed at recruitment using validated country or centre-specific dietary questionnaires^(20)^. All participants provided a written informed consent. Ethical approval for the EPIC study was obtained from the review boards of the International Agency for Research on Cancer (Lyon, France) and local participating centres.

### Laboratory methods and reporting

Plasma hsCRP concentrations were measured using a high-sensitivity assay (Beckman-Coulter) on a Synchron LX-20 Pro autoanalyser (Beckman-Coulter)^(18)^. The interassay CV were 6·0 and 6·5 % at hsCRP concentrations of 1·16 mg/l and 1·89 mg/l, respectively. Plasma polyphenol measurements for thirty-five compounds were performed using a highly sensitive method based on differential isotope labelling with (13)C- and (12)C-dansyl chloride by tandem MS^(21)^. Limits of quantification for the polyphenols varied between 0·11 nmol/l for apigenin and 44·4 nmol/l for quercetin. Intra-batch CV varied between 2·3 and 9·0 %. Inter-batch CV were <20 % for all except for quercetin, gallic acid, hydroxytyrosol and enterodiol.

### Statistical analysis

Differences in medians of hsCRP and polyphenol concentrations according to participant characteristics were assessed using Wilcoxon–Mann–Whitney test for dichotomous variables and Kruskal–Wallis test for variables with more than two categories. Participants with missing values in any of the polyphenol subclasses or hsCRP were excluded, while missing values in categorical adjustment variables were placed in a separate category.

Right-skewed data distributions were standardised using box-cox transformations. Values of plasmatic polyphenol concentrations were *z*-transformed for analysis according to standard deviations and back-transformed to natural units for presentation in text and tables. Several compounds, including gallocatechin, epigallocatechin, phloretin and gallic acid ethyl ester, were excluded from statistical analysis because of a too limited number of values above the limit of detection (<5 %). A variable ‘combined polyphenols’ was created based on the sum of plasma concentrations of all polyphenols measured in the study sample.

Geometric means and 95 % CI of hsCRP by plasma polyphenol concentrations were estimated using ANCOVA. Statistical tests for trend for a given polyphenol were calculated using the ordinal quartile entered into the models as a continuous variable. Covariates for the multivariable-adjusted analyses were chosen *a priori* based on reported associations with circulating hsCRP in the literature. The variable list included age, sex, country, education, smoking status, alcohol intake, red and processed meat consumption, fibre consumption, fish and shellfish intake, physical activity, BMI, waist circumference, prevalent diabetes and cardiovascular problems^(22–28)^. In logistic regression analysis, ‘elevated hsCRP’ was defined as response variable dichotomised based on established cut point of hsCRP ≥ 3 mg/l *v*. hsCRP <3 mg/l denoting individual chronic inflammatory status^(4)^. The OR and 95 % CI of elevated hsCRP were calculated within quartiles of polyphenols distribution and per sd increase of polyphenol concentrations. To test for non-linearity, we fitted restricted cubic splines, at the 10th, 50th and 90th percentiles of polyphenol concentrations, to the fully adjusted logistic regression models and used the Wald *χ*^2^ test.

To identify major dietary predictors of circulating polyphenol concentrations in our study sample, we applied a variable selection using adaptive least absolute shrinkage and selection operator regression model with ‘combined polyphenols’ as dependent variable and reported individual food intakes (*n* 212) as independent variables. Least absolute shrinkage and selection operator is a penalised regression method proven to outperform traditional regression methods (i.e. stepwise and forward selection) when there are correlated predictors or when the number of predictors is large as in our study. SBC was used as a tuning method to build a model using adaptive least absolute shrinkage and selection operator regression. As a next step, *β*-coefficients and 95 % CI between the variable ‘combined polyphenols’ and the identified best set of dietary predictors were calculated in linear regression analysis.

In sensitivity analyses, main associations were evaluated excluding participants with polyphenol concentrations in the highest and lowest percentile, women using hormone replacement therapy (*n* 22) and individuals whose waist circumference was imputed (*n* 11). Analyses were also repeated excluding participants with hsCRP ≥ 10 mg/l (*n* 18) potentially indicating acute inflammatory response. Statistical tests were considered to be significant when *P* < 0·05. All statistical analyses were performed in SAS (Version 9.4, Enterprise Guide 6.1, SAS Institute Inc.).

## Results

In the present study, sample hsCRP ranged from 0·20 to 23·16 mg/l. In total, 113 participants (36 % of the sample) had hsCRP ≥ 3 mg/l. Median hsCRP concentrations were higher in women as well as in participants with reported CVD and type 2 diabetes and higher BMI and waist circumference at study baseline compared with their counterparts (Table 1). hsCRP concentrations were lower in participants with medium to high fibre intake and high fish and shellfish intake (see Table 1). The relative proportion of polyphenol subclasses and individual compounds to the combined polyphenol variable is presented in online Supplementary Fig. S2A and S2B, respectively. Phenolic acids (75 %) and flavonoids (25 %) represented the largest share of polyphenol subclasses, whereas caffeic acid (16 %), 4-hydroxiphenylacetic acid (13 %) and quercetin (13 %) had greatest share among individual polyphenols.

**Table 1.** Serum high-sensitivity C-reactive protein (hsCRP) concentrations by participant characteristics (*n* 315) (Numbers and percentages; medians and 25th and 75th percentiles)

			Serum hsCRP (mg/l)			
Variable	*n*	%	Median	25th percentile	75th percentile	P*
Sex						
All	315	100·0	2·15	1·06	4·05	0·001
Male	116	36·8	1·65	0·76	3·19	
Female	199	63·2	2·56	1·17	4·43	
Age (years)						
<40	5	1·6	2·33	1·73	2·81	0·21
40–49	41	13·0	1·65	0·56	2·61
50–59	143	45·4	2·25	0·97	4·33
60–69	120	45·4	2·15	1·14	4·27
≥70	6	1·9	2·72	2·15	7·02
Highest school level						
Not specified	7	2·2	4·11	1·37	5·39	0·012
None	19	6·0	1·64	1·08	4·43
Primary school completed	123	39·0	2·62	1·44	5·16
Technical/professional school	69	21·9	1·77	0·76	3·17
Secondary school	47	14·9	2·42	1·09	3·84
Longer education	50	15·9	1·58	0·74	2·91
Diabetes mellitus						
Not specified	25	7·9	1·83	1·06	3·63	0·67
No	276	87·6	2·21	1·07	4·05
Yes	14	4·4	2·47	0·88	6·49
CVD						
Not specified	39	12·4	2·69	1·09	5·16	0·028
No	178	56·5	1·91	0·85	3·69
Yes	98	31·1	2·57	1·25	4·40
Smoking status						
Not specified	1	0·3	3·76	3·76	3·76	0·87
Never	150	47·6	2·15	0·97	3·85
Former	96	30·5	1·98	1·10	4·08
Current	68	21·6	2·24	1·14	4·07
Alcohol consumption (g/d)						
Non-drinkers	16	5·1	4·14	0·93	7·02	0·35
≤10	164	52·1	2·33	1·11	4·02
10–40	112	35·6	1·87	0·91	3·81
>40	23	7·3	2·19	1·31	3·90
Physical activity						
Not specified	13	4·1	2·85	1·09	3·85	0·78
Inactive	31	9·8	1·62	0·97	3·90
Moderately inactive	91	28·9	2·48	0·90	4·65
Moderately active	148	47·1	2·17	1·12	3·79
Active	32	10·2	1·89	0·81	4·41
BMI (kg/m^2^)						
<20	6	1·9	1·26	0·47	3·99	<0·001
20–24·9	112	35·6	1·77	0·66	2·90
25–29·9	151	47·9	2·31	1·11	4·24
≥30	46	14·6	3·14	1·83	5·65
Waist circumference (cm)						
Men						
<94	50	43·1	1·26	0·55	2·60	0·025
≥94	66	56·9	1·83	1·09	3·90
Women						
<80	87	43·7	1·79	0·85	3·54	<0·0001
≥80	112	56·3	3·17	1·81	4·72
Total energy intake (kJ/d)						
Men						
≤10 460	72	62·1	1·83	0·83	3·69	0·43
>10 460	44	37·9	1·52	0·76	2·63
Women						
≤8368	123	61·8	2·78	1·42	4·70	0·11
>8368	76	38·2	2·13	1·04	4·21
Total dietary fibre (g/d)						
≤20	119	37·8	2·66	1·62	5·00	<0·001
20–30	151	47·9	1·91	0·89	3·87
>30	45	14·3	1·50	0·73	2·56
Processed and red meat intake (g/d)						
≤50	80	25·4	1·90	1·21	3·13	0·17
50–150	210	66·7	2·39	1·01	4·72
>150	25	7·9	1·54	0·82	2·62
Fish and shellfish intake (g/d)						
Non-consumers	13	4·1	2·15	1·50	3·77	0·78
≤50	236	74·9	2·22	1·07	4·02
>50	66	21·0	1·96	1·02	4·05

* *P* values by Wilcoxon–Mann–Whitney test or Kruskal–Wallis test among subgroups for each variable.

Median values of combined polyphenols were higher in women and in participants free of CVD at study baseline (online Supplementary Table S1). No substantial differences were observed according to levels of physical activity, BMI and waist circumference categories, fish and shellfish intake and country of origin of EPIC participants (see online Supplementary Tables S1 and S2).

**Fig. 1.**
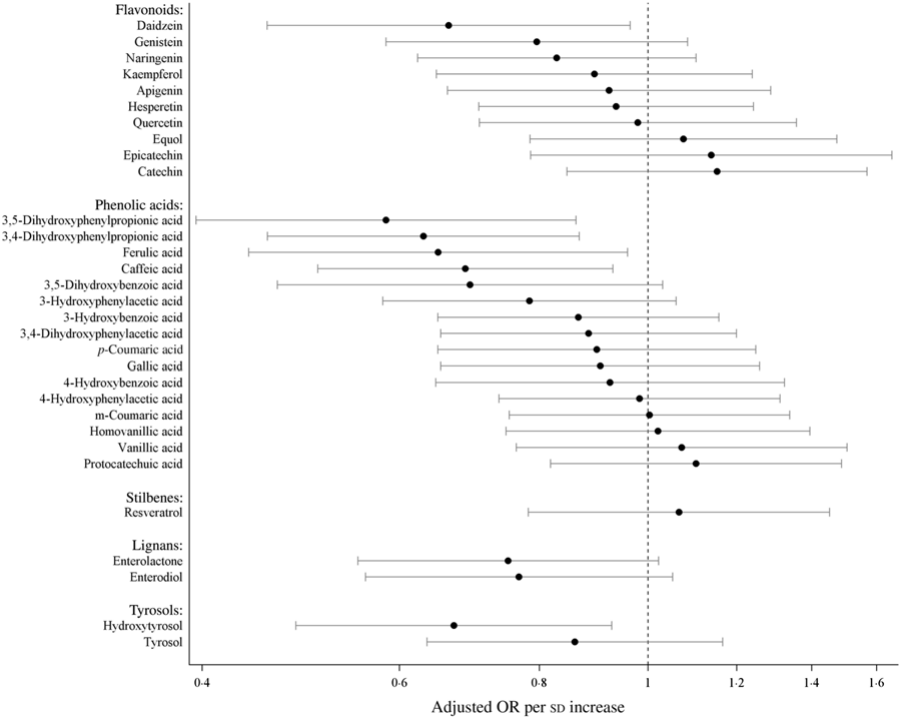
Risk for high-sensitivity C-reactive protein ≥ 3 mg/l per standard deviation increase of polyphenol concentrations. Models were adjusted for age, sex, country, diabetes, cardiovascular problems, education, smoking status, alcohol intake, red and processed meat consumption, total fibre consumption, fish and shellfish intake, total physical activity and BMI-adjusted waist circumference. Values are adjusted odds ratios, with 95 % confidence intervals represented by horizontal bars.

**Fig. 2.**
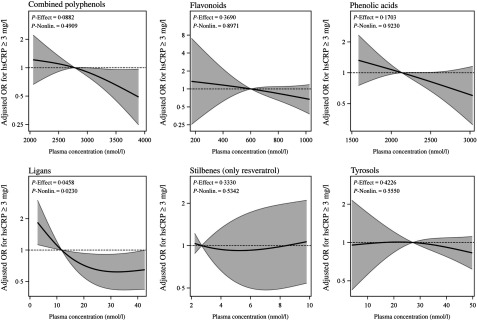
Odds ratios and 95 % confidence interval function for high-sensitivity C-reactive protein (hsCRP) ≥ 3 mg/l estimated by a restricted cubic spline function with three knots at the 10th, 50th and 90th percentile of concentrations of total polyphenols and polyphenol classes. Models were adjusted for age, sex, country, diabetes, cardiovascular problems, education, smoking status, alcohol intake, red and processed meat consumption, total fibre consumption, fish and shellfish intake, total physical activity and BMI-adjusted waist circumference. Nonlin., non-linear.

In multivariable-adjusted model, higher plasma concentrations of combined polyphenols (modelled continuously per sd higher concentrations) were associated with 29 (95 % CI 50, 1) % lower odds of elevated hsCRP (Table 2). Per sd higher concentration, the OR of elevated hsCRP were 0·71 (95 % CI 0·44, 1·15) for flavonoids, 0·74 (95 % CI 0·54, 1·02) for phenolic acids, 0·71 (95 % CI 0·52, 0·98) for lignans, 1·07 (0·78, 1·45) for stilbenes (resveratrol only) and 0·88 (95 % CI 0·66, 1·17) for tyrosols (Fig. 1). For the majority of polyphenol concentrations summed according to subclasses, the associations proved to be linear, with the exception of resveratrol (*P* for non-linearity > 0·05) (see Fig. 2). A more detailed inspection of analyses by quartiles of resveratrol showed that the OR for elevated hsCRP were 0·38 (95 % CI 0·15, 0·94), 0·86 (95 % CI 0·41, 1·82) and 0·79 (95 % CI 0·34, 1·83) in the second, third and fourth quartiles compared with the first quartile, respectively (Table 2). Several specific polyphenol compounds were statistically significantly associated with lower odds for elevated CRP (Fig. 2). Such associations were revealed for daidzein (flavonoid); ferulic acid, caffeic acid, 3,4-dihydroxyphenylpropionic acid and 3,5 dihydroxybenzoic acid (phenolic acids); enterolactone and enterodiol (lignans) and hydroxytyrosol (phenolic alcohol) (online Supplementary Table S3). In spline regression analysis, no pronounced deviation from linearity could be seen for associations with majority of individual polyphenol compounds (online Supplementary Fig. S3). Exceptions were the associations with enterolactone (*P* non-linearity = 0·028), 3,4-dihydroxyphenylpropionic acid (*P* non-linearity < 0·001) and *m*-coumaric acid (*P* non-linearity = 0·03).

**Table 2.** High-sensitivity C-reactive protein (hsCRP) concentrations and estimated risk for elevated hsCRP (>3 mg/l) according to quartiles (Q) of polyphenol concentrations and per standard deviation increase (Geometric mean values and 95 % confidence intervals; odds ratios and 95 % confidence intervals)

Polyphenol subclasses	Quartiles of polyphenol concentrations											
	Q1 (*n* 78)		Q2 (*n* 79)		Q3 (*n* 79)		Q4 (*n* 79)			Per sd increase of polyphenol concentrations		
	Mean	95 % CI	Mean	95 % CI	Mean	95 % CI	Mean	95 % CI	*P*_linear trend_	OR	95 % CI	*P*
Combined polyphenols												
hsCRP (mg/l)												
Geometric mean	2·34		1·76		2·15		1·80		–			
Model 1*†	2·32	1·81, 2·97	1·77	1·39, 2·27	1·99	1·58, 2·52	1·50	1·14, 1·97	0·12			
Model 2* ‡	2·23	1·70, 2·91	1·68	1·28, 2·20	1·75	1·34, 2·28	1·34	1·00, 1·79	0·069			
OR for hsCRP ≥3 mg/l^3^	1·00		1·16	0·52, 2·60	0·57	0·25, 1·29	0·42	0·16, 1·11	0·038	0·71	0·50, 0·99	0·045
Flavonoids												
hsCRP (mg/l)												
Geometric mean	2·12		2·23		1·77		1·91		–			
Model 1* †	2·02	1·40, 2·89	2·10	1·63, 2·71	1·85	1·43, 2·39	1·62	1·23, 2·15	0·51			
Model 2* ‡	1·88	1·29, 2·73	1·99	1·52, 2·61	1·71	1·29, 2·25	1·41	1·04, 1·91	0·24			
OR for hsCRP ≥3 mg/l^3^	1·00		0·86	0·26, 2·87	0·74	0·22, 2·53	0·43	0·12, 1·59	0·10	0·71	0·44, 1·15	0·17
Phenolic acids												
hsCRP (mg/l)												
Geometric mean	2·11		2·14		1·84		1·92		–			
Model 1* †	2·18	1·69, 2·81	2·10	1·65, 2·68	1·72	1·36, 2·17	1·65	1·27, 2·15	0·30			
Model 2* ‡	2·13	1·62, 2·79	1·91	1·47, 2·48	1·61	1·23, 2·09	1·40	1·06, 1·86	0·11			
OR for hsCRP ≥3 mg/l^3^	1·00		0·82	0·37, 1·85	0·63	0·28, 1·43	0·28	0·11, 0·72	0·008	0·74	0·54, 1·02	0·066
Lignans												
hsCRP (mg/l)												
Geometric mean	2·59		1·92		1·99		1·61		–			
Model 1*†	2·70	2·12, 3·45	1·86	1·46, 2·36	1·82	1·45, 2·29	1·45	1·14, 1·84	0·003			
Model 2* ‡	2·34	1·80, 3·04	1·75	1·33, 2·29	1·63	1·26, 2·09	1·41	1·07, 1·84	0·028			
OR for hsCRP ≥3 mg/l^3^	1·00		0·53	0·24, 1·14	0·42	0·18, 0·95	0·34	0·15, 0·79	0·012	0·71	0·52, 0·98	0·034
Stilbenes (resveratrol only)												
hsCRP (mg/l)												
Geometric mean	2·32		1·63		1·97		1·9		–			
Model 1* †	2·22	1·81, 2·72	1·46	1·09, 1·96	1·81	1·42, 2·31	1·78	1·38, 2·29	0·13			
Model 2* ‡	2·06	1·64, 2·60	1·35	0·99, 1·84	1·67	1·28, 2·19	1·61	1·20, 2·16	0·12			
OR for hsCRP ≥3 mg/l^3^	1·00		0·38	0·15, 0·94	0·86	0·41, 1·82	0·79	0·34, 1·83	0·95	1·07	0·78, 1·45	0·69
Tyrosols												
hsCRP (mg/l)												
Geometric mean	1·89		2·27		2·07		1·8		–			
Model 1* †	1·92	1·50, 2·45	2·16	1·69, 2·76	1·84	1·44, 2·34	1·75	1·38, 2·22	0·63			
Model 2* ‡	1·81	1·39, 2·35	1·85	1·40, 2·43	1·76	1·34, 2·32	1·64	1·27, 2·13	0·90			
OR for hsCRP ≥3 mg/l^3^	1·00		1·03	0·48, 2·25	0·80	0·36, 1·80	0·58	0·26, 1·31	0·15	0·88	0·66, 1·17	0·38

* Values are geometric means (*n* 315).

† Adjusted for age, sex, country and total energy intake

‡ Adjusted for age, sex, country, diabetes, cardiovascular problems, education, smoking status, alcohol intake, red and processed meat consumption, total fibre consumption, fish and shellfish intake, total physical activity and BMI-adjusted waist circumference.

The best subset of dietary predictors of combined plasma polyphenol concentrations estimated based on adapted least absolute shrinkage and selection operator regression model is shown in Table 3. The model explained overall 23·4 % of the variation in combined plasma polyphenol concentrations. In a linear regression model, based on the best subset in which each predictor was mutually adjusted for each other, significant positive associations were observed between plasma polyphenol concentrations and the following dietary intake variables: ‘Pasta-like cereal-based products (not 100 % cereal)’; ‘Sauces (not specified)’; ‘Tomato sauces’; ‘Kiwi’; ‘Tea’ and ‘Coffee’ (Table 3).

**Table 3.** Potential dietary predictors* of combined polyphenol concentrations (*β-*Coefficients and 95 % confidence intervals)

Parameter	*β*	95 % CI	*P*
Pasta-like cereal-based products (not 100 % cereal)†	63·2	9·5, 117·1	0·021
Sauces (not specified)‡	22·6	10·9, 34·3	0·0001
Tomato sauces	8·1	−0·13, 16·3	0·053
Kiwi	2·8	−0·01, 5·72	0·050
Tea	0·68	0·40, 0·96	0·001
Coffee	0·35	0·13, 0·57	0·002

* The set of dietary predictors was determined based on linear least absolute shrinkage and selection operator regression model with initial number of variables for reported dietary intakes based on European Prospective Investigation into Cancer and Nutrition FFQ (*n* 212). Analysis was stratified by the study centre.

† The foods within this grouped dietary intake variable include pasta-like cereal-based products such as quenelle, gnocchi and dumplings.

‡ The foods within this grouped dietary intake variable include sauces for pasta, sauces for vegetables, soya sauce, pesto, green sauce, gravy, curry sauce and peanut sauce.

In sensitivity analysis, excluding participants with polyphenol concentrations in the highest and lowest percentile, women using hormone replacement therapy (*n* 22) and individuals whose waist circumference was imputed (*n* 11) did not substantially affect main results (data not shown).

## Discussion

In this cross-sectional analysis embedded within the EPIC cohort, we characterised plasma concentrations of thirty-five polyphenols in relation to hsCRP taking into account various factors of potential influence. These analyses showed that high plasma polyphenol concentrations were associated with lower odds of elevated hsCRP. Among specific polyphenol compounds, the associations have been most pronounced for daidzein (flavonoid); ferulic acid, caffeic acid, 3,4-dihydroxyphenylpropionic acid and 3,5 dihydroxybenzoic acid (phenolic acids); enterolactone and enterodiol (lignans) and hydroxytyrosol (phenolic alcohol).

To the best of our knowledge, this is the first epidemiological study to characterise potential anti-inflammatory properties of multiple polyphenol compounds measured in human plasma in a population-based sample of diverse European populations characterised by high variation in food intakes. Previously, only two small cross-sectional studies explored correlations between CRP concentrations and individual polyphenols in blood mostly focusing on compounds associated with coffee and tea intakes. The first study conducted among Japanese healthy females (*n* 57) showed that plasma chlorogenic acid was inversely correlated with circulating CRP, whereas plasma total coffee polyphenol and plasma caffeic acid were weakly inversely associated with CRP^(14)^. The second study also conducted in generally healthy Japanese females (*n* 57) suggested that plasma total and individual catechins associated with green tea intake were weakly to moderately associated with C-reactive protein^(15)^. Comparison of our findings with data from these studies is hampered by the lower number of target compounds and differing analytical techniques.

So far, several randomised control trials explored effects of dietary interventions based on polyphenol-rich foods on CRP levels, thereby conducting measurements of plasma polyphenol concentrations at pre- and post-intervention period. Results from two randomised control trials conducted in German^(16)^ and Finnish^(13)^ study participants showed no evidence of correlation between relative changes in plasma flavanols (i.e. quercetin and kaempferol) and changes in CRP. In contrast, a randomised control trial that evaluated intervention with soya supplements showed a strong inverse correlation between changes in specific flavonoids (i.e. daidzein) and changes in CRP^(12)^. However, when polyphenol compounds such as hydroxytyrosol^(29)^ and daidzein^(30)^ were administered as dietary supplements in randomised control trial studies, no effect on CRP could be observed. The discrepancy between observational and experimental epidemiological studies may be explained by the fact that polyphenol extracts used in supplementation and fortification may lack the synergistic effects and health benefits of a diet naturally rich in polyphenols. Our data specifically pointed to polyphenol compounds that could be particularly bioactive exerting anti-inflammatory properties. Among these, daidzein has been known as one of the most common compounds within the subclass of isoflavones^(31)^. The chemical structure of isoflavones resembles the structure of oestrogens, and main food sources include soya and its processed products^(32)^. In our data, a strong anti-inflammatory link was further suggested for the cinnamic acid derivatives of phenolic acids, including 3,4-dihydroxyphenylpropionic acid, 3,5-dihydroxyphenylpropionic acid, caffeic acid and ferulic acid. Caffeic acid has been described as the most abundant phenolic acid which main source is coffee. Coffee contains an ester known as chlorogenic acid that is largely hydrolysed into caffeic acid in the gut^(33)^. However, caffeic acid also accounts for over 75 % of the total hydroxycinnamic acid content of fruits^(34)^. Major sources of caffeic acid include blueberries, kiwis, plums, cherries and apples, as well as specific herbs and spices^(44)^. 3,4-Dihydroxyphenylpropionic acid also known as dihydrocaffeic acid is a metabolite identified in human plasma after ingestion of caffeic acid^(36)^ but can also be formed from other polyphenols such as catechin present in foods andbeverages such as tea, cocoa and wine. Ferulic acid is the most abundant phenolic acid found in cereal grains mostly present in their outer parts^(34)^. Maize flour, whole-grain wheat, rice and oat flours are known as main dietary sources of ferulic acid. Coffee may represent another dietary source of ferulic acid concentration^(33)^. 3,5-Dihydroxyphenylpropionoic acid is a metabolite of alkylresorcinols, associated with whole-grain wheat intake^(35)^. Tyrosol (4-hydroxyphenylethanol) and hydroxytyrosol (3,4-dihydroxyphenylethanol) are the main phenolic alcohols contained mainly in extra virgin olive oil but are also present in red and white wines and beer^(36)^. In particular, hydroxytyrosol is found in red wine and is additionally produced *in vivo* after red wine ingestion^(36)^. Finally, our analysis pointed to anti-inflammatory properties of enterolactone and enterodiol representing the class of lignans. They are formed from dietary lignans found in relatively low concentrations in various seeds, grains, fruits and vegetables and in higher concentrations in sesame and flax seeds^(37)^. They have been widely studied for their oestrogenic properties and were defined for this reason as phyto-oestrogens. Interestingly, our analysis revealed a specific J-shaped association between resveratrol and inflammatory status such that moderate resveratrol levels were associated with lower odds for elevated hsCRP. In contrast, very low levels and very high levels of resveratrol have been found associated with elevated inflammation levels. This finding could provide a curious parallel with the known J-shaped association for wine consumption and health outcomes^(38)^. Indeed, moderate wine consumption is a characteristic of the Mediterranean diet, and studies around the world have shown a beneficial effect of moderate wine on human health^(39)^. Whether consuming moderate amounts of resveratrol could provide a key for achieving optimal inflammatory state and lower risk of chronic diseases should be further evaluated. Overall, our data add to the increasing line of evidence from basic research on anti-inflammatory properties of polyphenols. Potential mechanisms explaining this link include (a) acting as an antioxidant or increasing antioxidant gene or protein expression, (b) attenuating endoplasmic reticulum stress signalling, (c) blocking pro-inflammatory cytokines or endotoxin-mediated kinases and transcription factors involved in metabolic disease, (d) suppressing inflammatory or inducing metabolic-gene expression via increasing histone deacetylase activity and (e) activating transcription factors that antagonise chronic inflammation^(40)^. More specifically, polyphenols that manifested an inverse association with hsCRP in our data, that is, daidzein, caffeic acid and its derivatives, enterolactone and enterodiol and hydroxityrosol, were shown to suppress the production of pro-inflammatory mediators by inhibiting their activity or gene expression through down-regulation of transcriptional factors such as NF-κB^(41–46)^. Enterolactone and enterodiol were also shown to pass the intestinal barrier and directly modulate cytokine production^(44)^, whereas hydroxytyrosol was further suggested to exacerbate improvement in the antioxidant potential of plasma^(46)^.

It should also be noted that the metabolites present in blood circulation result from digestive and hepatic activity and supposedly differ from the native compounds, and the complex interaction with individual gut microbiota and metabolism should be taken into account when interpreting human study data^(47)^. The bioavailability may differ greatly among the various polyphenol compounds, and the most abundant ones in human diet would not be necessarily those that have the best bioavailability profile. Nevertheless, high plasma concentrations of polyphenol metabolites could reflect regular and frequent consumption of plant products. Based on dietary data collected in the EPIC cohort, main foods that predicted concentrations of combined polyphenols included specific pasta-like cereal-based products and sauces (i.e. soya sauce and tomato sauce), coffee and tea. Among fruits, only kiwi was retained in the model. A polyphenol-rich dietary pattern with dense bioactive nutrient composition could have strong anti-inflammatory effect, and further methodological work would be warranted to develop and evaluate preventive potential of such a dietary approach.

A major strength of our study is the comparatively large number of polyphenols investigated spanning all major classes found in the diet. We were able to simultaneously quantify concentrations of thirty-five polyphenols by applying a newly developed analytical method^(21)^. The measurement of plasma concentrations of polyphenols represents a snapshot of internal exposure to these compounds that could originate from several dietary sources directly or their precursors. Thus, any potential bias using exposure measurements from questionnaire-based data acquisition is circumvented. Another strength of our study is that, compared with previous studies, we considered a large variety of covariates in the association of plasma polyphenols and hsCRP. Further, we were able to explore associations across study subjects of different lifestyle and dietary habits in nine different countries. As compared with characteristics of the full EPIC cohort, no indication of selection bias could be seen^(17)^. The key limitation of our study is its cross-sectional design, which precludes making inferences regarding causality. Furthermore, because of the observational nature of the study, the possibility of residual confounding cannot be avoided. Both polyphenols and hsCRP were measured in single plasma samples from baseline, meaning that intra-individual variations in circulating concentrations of these biomarkers were unaccounted for^(34)^. hsCRP concentrations, on the other hand, have been shown to be relatively stable in previous studies of non-diseased people, with an intra-class correlation coefficient of 0·67 over a 4-year period^(48)^. The variability in these measures could have biased the results towards the null. Our results are restricted to the measured polyphenol compounds and do not provide full picture on full polyphenol metabolome.

In summary, the present study revealed that high plasma polyphenol concentrations were associated with lower odds of elevated hsCRP. Among specific polyphenol compounds, the associations have been most pronounced for daidzein, ferulic acid, caffeic acid, 3,4-dihydroxyphenylpropionic acid and 3,5 dihydroxybenzoic acid, enterolactone, enterodiol and hydroxityrosol. Whether diet rich in polyphenol compounds could be an effective strategy to prevent or modulate deleterious health effects of inflammation should be addressed by further well-powered longitudinal studies.

## Data Availability

For information on how to submit an application for gaining access to EPIC data and/or biospecimens, please follow the instructions at http://epic.iarc.fr/access/index.php

## Supplementary material

For supplementary material accompanying this paper visit https://doi.org/10.1017/S0007114519002538. click here to view supplementary material
