# Supplementary material for: Plasma polyphenols associated with lower high-sensitivity C-reactive protein concentrations: a cross-sectional study within the European Prospective Investigation into Cancer and Nutrition (EPIC) cohort
Source: Br J Nutr. 2020 Jan 28;123(2):198–208. doi: 10.1017/S0007114519002538 (PMC7015881; doi:10.1017/S0007114519002538)
Supplement: Supplementary file 1 [file S0007114519002538sup001.zip › S0007114519002538supp004.docx]

| **Supplementary Table 1.** Plasma concentrations of total polyphenols by participant characteristics | | | | | | | |
| --- | --- | --- | --- | --- | --- | --- | --- |
|  | | |  | Adjusted^1^ total polyphenols | | |  |
| Variable | | | N (%) | Median | Q1 | Q3 | P-value^2^ |
| Sex | | |  |  |  |  |  |
|  | All  Male  Female | | 315 (100.0)  116 (36.8)  199 (63.2) | -114.65  -151.67  -97.44 | -560.61  -488.43  -600.22 | 466.66  434.64  467.01 | **0.001** |
| Age (years) | | |  |  |  |  |  |
|  | 30-39  40-49  50-59  60-69  70-79 | | 5 (1.6)  41 (13.0)  143 (45.4)  120 (38.1)  6 (1.9) | -153.21  -528.79  -236.00  103.34  57.31 | -202.15  -741.46  -617.43  -380.70  -97.44 | -73.62  98.72  291.80  729.03  667.86 | **<0.0001** |
| Highest school level | | |  |  |  |  |  |
|  | Not specified  None  Primary school completed  Technical/professional school  Secondary school  Longer education | | 7 (2.2)  19 (6.0)  123 (39.0)  69 (21.9)  47 (14.9)  50 (15.9) | 235.71  -33.70  -157.22  -153.21  -82.47  -76.94 | -497.43  -643.81  -617.43  -624.62  -575.27  -455.31 | 891.45  243.64  479.51  332.11  732.01  467.01 | 0.80 |
| Diabetes mellitus | | |  |  |  |  |  |
|  | Not specified  No  Yes | | 25 (7.9)  276 (87.6)  14 (4.4) | 449.05  -155.22  -11.30 | -319.45  -620.43  -549.91 | 918.95  401.49  118.75 | 0.74 |
| Cardiovascular problems | | |  |  |  |  |  |
|  | Not specified  No  Yes | | 39 (12.4)  178 (56.5)  98 (31.1) | 207.05  449.24  35.52 | -450.97  -641.21  -480.14 | 644.57  260.55  699.29 | **0.012** |
| Smoking status | | |  |  |  |  |  |
|  | Not specified  Never  Former  Current | | 1 (0.3)  150 (47.6)  96 (30.5)  68 (21.6) | 216.44  -89.95  25.96  -257.17 | 216.44  -617.43  -468.51  -570.53 | 216.44  401.73  715.10  259.37 | 0.27 |
| Alcohol (g/day) | | |  |  |  |  |  |
|  | Non-drinkers  ≤ 10  >10-40  >40 | | 16 (5.1)  164 (52.1)  112 (35.6)  23 (7.3) | -270.82  -67.65  -112.00  -319.28 | -516.11  -606.17  489.95  -633.12 | 130.80  601.29  355.96  479.51 | 0.79 |
| Physical activity | | |  |  |  |  |  |
|  | Not specified  Inactive  Moderately inactive  Moderately active  Active | | 13 (4.1)  31 (9.8)  91 (28.9)  148 (47.1)  32 (10.2) | -658.86  -140.56  -113.57  -106.05  186.89 | -917.80  -485.51  -517.54  -565.18  -448.46 | -521.00  162.49  498.60  517.64  627.13 | 0.52 |
| BMI (kg/m^2^) | | |  |  |  |  |  |
|  | <20  20-24.9  25-29.9  ≥30 | | 6 (1.9)  112 (35.6)  151 (47.9)  46 (14.6) | -301.54  -78.04  -92.19  -238.71 | -797.83  -467.03  -528.79  -633.38 | 195.68  568.04  467.01  243.64 | 0.36 |
| Waist circumference (cm) | | |  |  |  |  |  |
|  | Men | |  |  |  |  |  |
|  |  | < 94  ≥ 94 | 50 (43.1)  66 (56.9) | -105.91  -219.14 | -415.63  -644.15 | 479.51  401.25 | 0.23 |
|  | Women | |  |  |  |  |  |
|  |  | < 80  ≥ 80 | 87 (43.7)  112 (56.3) | -54.29  -153.15 | -480.23  -621.02 | 594.38  290.04 | 0.20 |
| Total dietary fibre (g/day) | | |  |  |  |  |  |
|  | ≤ 20  > 20-30  > 30 | | 119 (37.8)  151 (47.9)  45 (14.3) | -157.22  -61.69  -97.44 | -639.23  -510.30  -525.64 | 538.42  467.01  332.11 | 0.75 |
| Processed and red meat intake (g/day) | | | |  |  |  |  |
|  | ≤ 50  > 50-150  > 150 | | 80 (25.4)  210 (66.7)  25 (7.9) | -48.30  -179.94  3.32 | -570.72  -575.27  -415.63 | 698.70  401.73  337.19 | 0.63 |
| Fish and shellfish intake (g/day) | | |  |  |  |  |  |
|  | Non-consumers  ≤ 50  > 50 | | 13 (4.1)  236 (74.9)  66 (21.0) | 760.57  -128.14  -251.18 | 7.04  -523.32  -691.14 | 1687.59  473.26  234.27 | **<0.001** |

^1^ Total plasma polyphenols were adjusted for total energy intake with the residual method
^2^ P-values by Wilcoxon-Mann-Whitney test or Kruskal-Wallis test among subgroups for each variable.
Abbreviations: Q1, First quartile value; Q3, Third quartile value.
