# Supplementary material for: Plasma polyphenols associated with lower high-sensitivity C-reactive protein concentrations: a cross-sectional study within the European Prospective Investigation into Cancer and Nutrition (EPIC) cohort
Source: Br J Nutr. 2020 Jan 28;123(2):198–208. doi: 10.1017/S0007114519002538 (PMC7015881; doi:10.1017/S0007114519002538)
Supplement: Supplementary file 1 [file S0007114519002538sup001.zip › S0007114519002538supp005.docx]

| **Supplementary Table 2.** Plasma concentrations of combined polyphenols by country | | | | | |
| --- | --- | --- | --- | --- | --- |
|  |  | Adjusted^1^ total polyphenols | | | |
| Country | n | Median | Q1 | Q3 | P-value^2^ |
| France  Italy  Spain  United Kingdom  The Netherlands  Greece  Germany  Sweden  Denmark | 15  62  41  28  50  10  47  11  51 | -273.76  -216.87  -460.71  645.10  573.85  -15.14  178.88  -882.92  -455.31 | -783.63  -617.43  -694.29  147.29  102.48  560.61  -345.87  -1072.69  -747.25 | 162.49  110.93  -129.72  1255.23  999.95  243.64  597.39  -528.79  72.94 | **<0.0001** |

^1^ Total plasma polyphenols were adjusted for total energy intake with the residual method.
^2^ P-values by Kruskal-Wallis test
Abbreviations: Q1, First quartile value; Q3, Third quartile value.
