# Supplementary material for: Plasma polyphenols associated with lower high-sensitivity C-reactive protein concentrations: a cross-sectional study within the European Prospective Investigation into Cancer and Nutrition (EPIC) cohort
Source: Br J Nutr. 2020 Jan 28;123(2):198–208. doi: 10.1017/S0007114519002538 (PMC7015881; doi:10.1017/S0007114519002538)
Supplement: Supplementary file 1 [file S0007114519002538sup001.zip › S0007114519002538supp006.docx]

| **Supplementary Table 3**. C-reactive protein concentration by plasma concentrations of individual polyphenol compounds | | | | | | | | | | | |
| --- | --- | --- | --- | --- | --- | --- | --- | --- | --- | --- | --- |
|  | | | Quartiles of polyphenol concentrations | | | | | |  | Per SD increase of polyphenol concentrations^4^ | |
| Polyphenol classes | | | Q1 | | Q2 | Q3 | Q4 | P-value for linear trend |  | OR (95% CI) | P-value |
| **Flavonoids** | | |  | |  |  |  |  |  |  |  |
| Kaempferol | | |  | |  |  |  |  |  |  |  |
|  | n | | 82 | | 83 | 73 | 77 |  |  |  |  |
|  | CRP, mg/L | |  | |  |  |  |  |  |  |  |
|  |  | Geometric mean | 2.08 | | 2.14 | 1.95 | 1.82 | - |  |  |  |
|  |  | Model 1^1,2^ | 1.98 (1.53-2.57) | | 2.12 (1.68-2.69) | 1.93 (1.52-2.45) | 1.58 (1.22-2.06) | 0.38 |  |  |  |
|  |  | Model 2^1,3^ | 1.83 (1.38-2.42) | | 1.99 (1.55-2.55) | 1.74 (1.33-2.27) | 1.43 (1.07-1.90) | 0.28 |  |  |  |
|  | OR for CRP ≥3 mg/L^3^ | | 1.00 | | 1.24 (0.57-2.68) | 0.49 (0.21-1.15) | 0.62 (0.26-1.51) | 0.098 |  | 0.90 (0.65-1.24) | 0.51 |
| Quercetin | | |  | |  |  |  |  |  |  |  |
|  | n | | 63 | | 63 | 63 | 64 |  |  |  |  |
|  | CRP, mg/L | |  | |  |  |  |  |  |  |  |
|  |  | Geometric mean | 2.10 | | 1.67 | 2.20 | 1.87 | - |  |  |  |
|  |  | Model 1^1,2^ | 2.05 (1.57-2.68) | | 1.61 (1.25-2.06) | 2.27 (1.74-2.95) | 1.65 (1.25-2.17) | 0.16 |  |  |  |
|  |  | Model 2^1,3^ | 2.13 (1.61-2.81) | | 1.65 (1.26-2.15) | 2.18 (1.66-2.87) | 1.62 (1.22-2.16) | 0.23 |  |  |  |
|  | OR for CRP ≥3 mg/L^3^ | | 1.00 | | 0.55 (0.22-1.34) | 1.15 (0.48-2.75) | 0.55 (0.22-1.37) | 0.48 |  | 0.98 (0.71-1.36) | 0.90 |
| Catechin | | |  | |  |  |  |  |  |  |  |
|  | n | | 78 | | 81 | 76 | 79 |  |  |  |  |
|  | CRP, mg/L | |  | |  |  |  |  |  |  |  |
|  |  | Geometric mean | 2.06 | | 2.11 | 1.78 | 2.09 | - |  |  |  |
|  |  | Model 1^1,2^ | 2.00 (1.57-2.54) | | 1.97 (1.55-2.50) | 1.74 (1.34-2.24) | 1.98 (1.53-2.56) | 0.81 |  |  |  |
|  |  | Model 2^1,3^ | 1.75 (1.34-2.29) | | 1.84 (1.41-2.39) | 1.54 (1.15-2.07) | 1.87 (1.41-2.46) | 0.65 |  |  |  |
|  | OR for CRP ≥3 mg/L^3^ | | 1.00 | | 1.75 (0.80-3.84) | 0.96 (0.39-2.33) | 1.59 (0.65-3.86) | 0.54 |  | 1.15 (0.85-1.57) | 0.37 |
| Epicatechin | | |  | |  |  |  |  |  |  |  |
|  | n | | 78 | | 79 | 79 | 79 |  |  |  |  |
|  | CRP, mg/L | |  | |  |  |  |  |  |  |  |
|  |  | Geometric mean | 2.11 | | 1.82 | 2.32 | 1.79 | - |  |  |  |
|  |  | Model 1^1,2^ | 2.09 (1.63-2.68) | | 1.8 (1.39-2.31) | 2.19 (1.71-2.81) | 1.57 (1.19-2.07) | 0.21 |  |  |  |
|  |  | Model 2^1,3^ | 1.75 (1.32-2.31) | | 1.59 (1.21-2.09) | 2.06 (1.57-2.70) | 1.68 (1.24-2.27) | 0.40 |  |  |  |
|  | OR for CRP ≥3 mg/L^3^ | | 1.00 | | 1.24 (0.56-2.76) | 1.87 (0.79-4.46) | 1.42 (0.52-3.87) | 0.34 |  | 1.14 (0.79-1.65) | 0.49 |
| Apigenin | | |  | |  |  |  |  |  |  |  |
|  | n | | 79 | | 83 | 75 | 78 |  |  |  |  |
|  | CRP, mg/L | |  | |  |  |  |  |  |  |  |
|  |  | Geometric mean | 2.17 | | 2.03 | 1.62 | 2.21 | - |  |  |  |
|  |  | Model 1^1,2^ | 2.06 (1.53-2.78) | | 1.94 (1.54-2.45) | 1.52 (1.17-1.98) | 2.08 (1.61-2.68) | 0.23 |  |  |  |
|  |  | Model 2^1,3^ | 1.79 (1.31-2.44) | | 1.74 (1.34-2.26) | 1.62 (1.22-2.14) | 1.98 (1.48-2.65) | 0.66 |  |  |  |
|  | OR for CRP ≥3 mg/L^3^ | | 1.00 | | 1.03 (0.4-2.67) | 0.41 (0.14-1.15) | 0.83 (0.31-2.25) | 0.44 |  | 0.92 (0.66-1.29) | 0.64 |
| Naringenin | | |  | |  |  |  |  |  |  |  |
|  | n | | 82 | | 78 | 76 | 79 |  |  |  |  |
|  | CRP, mg/L | |  | |  |  |  |  |  |  |  |
|  |  | Geometric mean | 1.81 | | 1.91 | 2.35 | 1.97 | - |  |  |  |
|  |  | Model 1^1,2^ | 1.79 (1.42-2.24) | | 1.82 (1.42-2.32) | 2.19 (1.71-2.81) | 1.89 (1.49-2.41) | 0.60 |  |  |  |
|  |  | Model 2^1,3^ | 1.67 (1.31-2.14) | | 1.76 (1.34-2.32) | 1.93 (1.46-2.55) | 1.76 (1.36-2.29) | 0.86 |  |  |  |
|  | OR for CRP ≥3 mg/L^3^ | | 1.00 | | 1.04 (0.47-2.29) | 1.15 (0.52-2.52) | 0.87 (0.39-1.91) | 0.78 |  | 0.83 (0.62-1.10) | 0.20 |
| Hesperetin | | |  | |  |  |  |  |  |  |  |
|  | n | | 78 | | 79 | 79 | 79 |  |  |  |  |
|  | CRP, mg/L | |  | |  |  |  |  |  |  |  |
|  |  | Geometric mean | 1.86 | | 1.8 | 2.32 | 2.05 | - |  |  |  |
|  |  | Model 1^1,2^ | 1.80 (1.41-2.30) | | 1.87 (1.47-2.36) | 2.10 (1.65-2.67) | 1.86 (1.44-2.38) | 0.79 |  |  |  |
|  |  | Model 2^1,3^ | 1.67 (1.29-2.18) | | 1.66 (1.27-2.18) | 2.02 (1.56-2.62) | 1.66 (1.26-2.19) | 0.55 |  |  |  |
|  | OR for CRP ≥3 mg/L^3^ | | 1.00 | | 1.05 (0.47-2.35) | 1.27 (0.59-2.73) | 1.02 (0.46-2.27) | 0.82 |  | 0.94 (0.71-1.24) | 0.65 |
| Daidzein | | |  | |  |  |  |  |  |  |  |
|  | n | | 68 | | 71 | 66 | 69 |  |  |  |  |
|  | CRP, mg/L | |  | |  |  |  |  |  |  |  |
|  |  | Geometric mean | 2.14 | | 2.1 | 1.74 | 2.01 | - |  |  |  |
|  |  | Model 1^1,2^ | 2.21 (1.7-2.89) | | 2.19 (1.71-2.81) | 1.78 (1.36-2.32) | 1.76 (1.34-2.32) | 0.50 |  |  |  |
|  |  | Model 2^1,3^ | 2.14 (1.6-2.85) | | 1.91 (1.46-2.49) | 1.83 (1.37-2.44) | 1.58 (1.17-2.14) | 0.52 |  |  |  |
|  | OR for CRP ≥3 mg/L^3^ | | 1.00 | | 1.04 (0.46-2.37) | 0.74 (0.29-1.88) | 0.43 (0.16-1.18) | 0.084 |  | 0.66 (0.46-0.96) | **0.031** |
| Genistein | | |  | |  |  |  |  |  |  |  |
|  | n | | 81 | | 76 | 79 | 79 |  |  |  |  |
|  | CRP, mg/L | |  | |  |  |  |  |  |  |  |
|  |  | Geometric mean | 1.97 | | 2.21 | 1.92 | 1.91 | - |  |  |  |
|  |  | Model 1^1,2^ | 1.82 (1.43-2.32) | | 2.25 (1.77-2.87) | 1.80 (1.41-2.30) | 1.76 (1.36-2.28) | 0.43 |  |  |  |
|  |  | Model 2^1,3^ | 1.72 (1.32-2.24) | | 2.06 (1.58-2.70) | 1.69 (1.30-2.21) | 1.56 (1.17-2.07) | 0.41 |  |  |  |
|  | OR for CRP ≥3 mg/L^3^ | | 1.00 | | 0.91 (0.42-2.01) | 0.88 (0.39-1.98) | 0.50 (0.21-1.21) | 0.15 |  | 0.80 (0.58-1.08) | 0.15 |
| Equol | | |  | |  |  |  |  |  |  |  |
|  | n | | 85 | | 59 | 71 | 70 |  |  |  |  |
|  | CRP, mg/L | |  | |  |  |  |  |  |  |  |
|  |  | Geometric mean | 1.61 | | 2.28 | 1.89 | 2.49 | - |  |  |  |
|  |  | Model 1^1,2^ | 1.55 (1.21-1.97) | | 2.18 (1.64-2.91) | 1.91 (1.46-2.51) | 2.45 (1.89-3.17) | **0.043** |  |  |  |
|  |  | Model 2^1,3^ | 1.45 (1.07-1.95) | | 1.87 (1.36-2.58) | 1.71 (1.26-2.32) | 2.19 (1.64-2.92) | 0.12 |  |  |  |
|  | OR for CRP ≥3 mg/L^3^ | | 1.00 | | 1.09 (0.45-2.63) | 0.91 (0.38-2.21) | 1.73 (0.74-4.09) | 0.25 |  | 1.08 (0.78-1.47) | 0.65 |
| **Phenolic acids** | | |  | |  |  |  |  |  |  |  |
| 4-Hydroxybenzoic acid | | |  | |  |  |  |  |  |  |  |
|  | n | | 68 | | 68 | 67 | 67 |  |  |  |  |
|  | CRP, mg/L | |  | |  |  |  |  |  |  |  |
|  |  | Geometric mean | 2.13 | | 1.99 | 1.89 | 2 | - |  |  |  |
|  |  | Model 1^1,2^ | 2.31 (1.77-3.03) | | 1.97 (1.52-2.55) | 1.74 (1.35-2.24) | 1.66 (1.27-2.17) | 0.31 |  |  |  |
|  |  | Model 2^1,3^ | 2.08 (1.56-2.79) | | 1.82 (1.37-2.42) | 1.61 (1.22-2.13) | 1.51 (1.13-2.00) | 0.39 |  |  |  |
|  | OR for CRP ≥3 mg/L^3^ | | 1.00 | | 1.42 (0.61-3.28) | 1.41 (0.57-3.51) | 0.67 (0.25-1.78) | 0.43 |  | 0.93 (0.65-1.32) | 0.67 |
| 3-Hydroxybenzoic acid | | |  | |  |  |  |  |  |  |  |
|  | n | | 78 | | 78 | 78 | 79 |  |  |  |  |
|  | CRP, mg/L | |  | |  |  |  |  |  |  |  |
|  |  | Geometric mean | 1.92 | | 2.62 | 1.6 | 1.99 | - |  |  |  |
|  |  | Model 1^1,2^ | 1.83 (1.45-2.31) | | 2.41 (1.91-3.05) | 1.53 (1.2-1.95) | 1.77 (1.38-2.27) | **0.039** |  |  |  |
|  |  | Model 2^1,3^ | 1.64 (1.27-2.11) | | 2.29 (1.77-2.96) | 1.43 (1.09-1.87) | 1.64 (1.26-2.13) | **0.022** |  |  |  |
|  | OR for CRP ≥3 mg/L^3^ | | 1.00 | | 1.64 (0.76-3.53) | 0.65 (0.27-1.52) | 0.89 (0.40-2.00) | 0.34 |  | 0.87 (0.65-1.16) | 0.33 |
| Protocatechuic acid | | |  | |  |  |  |  |  |  |  |
|  | n | | 79 | | 78 | 81 | 77 |  |  |  |  |
|  | CRP, mg/L | |  | |  |  |  |  |  |  |  |
|  |  | Geometric mean | 1.96 | | 1.8 | 2.07 | 2.17 | - |  |  |  |
|  |  | Model 1^1,2^ | 1.94 (1.53-2.46) | | 1.78 (1.40-2.27) | 1.92 (1.52-2.43) | 1.98 (1.53-2.57) | 0.93 |  |  |  |
|  |  | Model 2^1,3^ | 1.84 (1.42-2.38) | | 1.68 (1.29-2.19) | 1.68 (1.29-2.18) | 1.86 (1.41-2.47) | 0.87 |  |  |  |
|  | OR for CRP ≥3 mg/L^3^ | | 1.00 | | 0.79 (0.35-1.76) | 0.95 (0.44-2.05) | 0.93 (0.41-2.11) | 0.99 |  | 1.10 (0.82-1.49) | 0.52 |
| Gallic acid | | |  | |  |  |  |  |  |  |  |
|  | n | | 82 | | 77 | 77 | 79 |  |  |  |  |
|  | CRP, mg/L | |  | |  |  |  |  |  |  |  |
|  |  | Geometric mean | 1.87 | | 2.17 | 2.17 | 1.82 | - |  |  |  |
|  |  | Model 1^1,2^ | 1.90 (1.48-2.45) | | 2.16 (1.68-2.78) | 1.99 (1.57-2.53) | 1.63 (1.25-2.11) | 0.47 |  |  |  |
|  |  | Model 2^1,3^ | 1.75 (1.34-2.30) | | 1.84 (1.38-2.44) | 1.89 (1.46-2.44) | 1.58 (1.19-2.10) | 0.76 |  |  |  |
|  | OR for CRP ≥3 mg/L^3^ | | 1.00 | | 1.09 (0.50-2.35) | 1.10 (0.51-2.38) | 0.85 (0.35-2.10) | 0.83 |  | 0.91 (0.65-1.26) | 0.56 |
| Vanillic acid | | |  | |  |  |  |  |  |  |  |
|  | n | | 80 | | 79 | 78 | 78 |  |  |  |  |
|  | CRP, mg/L | |  | |  |  |  |  |  |  |  |
|  |  | Geometric mean | 2.22 | | 1.90 | 1.93 | 1.95 | - |  |  |  |
|  |  | Model 1^1,2^ | 2.05 (1.59-2.64) | | 1.81 (1.42-2.32) | 1.85 (1.44-2.37) | 1.87 (1.45-2.43) | 0.90 |  |  |  |
|  |  | Model 2^1,3^ | 2.04 (1.55-2.69) | | 1.59 (1.21-2.10) | 1.61 (1.22-2.13) | 1.72 (1.31-2.25) | 0.49 |  |  |  |
|  | OR for CRP ≥3 mg/L^3^ | | 1.00 | | 0.66 (0.29-1.49) | 0.72 (0.31-1.64) | 0.64 (0.26-1.60) | 0.41 |  | 1.07 (0.76-1.51) | 0.69 |
| 3,5-Dihydroxybenzoic acid | | | | |  |  |  |  |  |  |  |
|  | n | | 78 | | 79 | 79 | 79 |  |  |  |  |
|  | CRP, mg/L | |  | |  |  |  |  |  |  |  |
|  |  | Geometric mean | 1.86 | | 2.18 | 2.15 | 1.83 | - |  |  |  |
|  |  | Model 1^1,2^ | 1.93 (1.45-2.57) | | 2.05 (1.63-2.59) | 1.95 (1.51-2.52) | 1.66 (1.26-2.17) | 0.66 |  |  |  |
|  |  | Model 2^1,3^ | 1.89 (1.39-2.56) | | 1.75 (1.36-2.25) | 1.75 (1.33-2.32) | 1.60 (1.17-2.19) | 0.89 |  |  |  |
|  | OR for CRP ≥3 mg/L^3^ | | 1.00 | | 0.94 (0.40-2.20) | 0.75 (0.29-1.95) | 0.57 (0.19-1.69) | 0.26 |  | 0.69 (0.47-1.03) | 0.070 |
| 4-Hydroxyphenylacetic acid | | | | |  |  |  |  |  |  |  |
|  | n | | | 77 | 76 | 76 | 77 |  |  |  |  |
|  | CRP, mg/L | | |  |  |  |  |  |  |  |  |
|  |  | Geometric mean | | 1.85 | 2.08 | 1.92 | 2.19 | - |  |  |  |
|  |  | Model 1^1,2^ | | 1.80 (1.41-2.29) | 1.96 (1.53-2.50) | 1.89 (1.47-2.44) | 1.92 (1.49-2.47) | 0.96 |  |  |  |
|  |  | Model 2^1,3^ | | 1.71 (1.32-2.22) | 1.76 (1.34-2.31) | 1.85 (1.41-2.43) | 1.71 (1.30-2.25) | 0.96 |  |  |  |
|  | OR for CRP ≥3 mg/L^3^ | | | 1.00 | 1.13 (0.52-2.45) | 1.10 (0.49-2.46) | 1.08 (0.48-2.43) | 0.87 |  | 0.98 (0.74-1.31) | 0.91 |
| 3-Hydroxyphenylacetic acid | | | | |  |  |  |  |  |  |  |
|  | n | | | 75 | 75 | 75 | 75 |  |  |  |  |
|  | CRP, mg/L | | |  |  |  |  |  |  |  |  |
|  |  | Geometric mean | | 2.31 | 2.08 | 1.71 | 2.12 | - |  |  |  |
|  |  | Model 1^1,2^ | | 2.32 (1.81-2.97) | 2.10 (1.64-2.69) | 1.62 (1.27-2.05) | 1.83 (1.42-2.36) | 0.16 |  |  |  |
|  |  | Model 2^1,3^ | | 2.04 (1.56-2.68) | 2.00 (1.52-2.64) | 1.54 (1.19-1.99) | 1.68 (1.26-2.24) | 0.30 |  |  |  |
|  | OR for CRP ≥3 mg/L^3^ | | | 1.00 | 0.90 (0.41-1.97) | 0.55 (0.24-1.26) | 0.71 (0.31-1.61) | 0.28 |  | 0.78 (0.58-1.06) | 0.11 |
| 3,4-Dihydroxyphenylacetic acid | | | | |  |  |  |  |  |  |  |
|  | n | | | 73 | 74 | 73 | 74 |  |  |  |  |
|  | CRP, mg/L | | |  |  |  |  |  |  |  |  |
|  |  | Geometric mean | | 2.3 | 2.23 | 2.09 | 1.67 | - |  |  |  |
|  |  | Model 1^1,2^ | | 2.33 (1.79-3.04) | 2.07 (1.63-2.64) | 1.92 (1.50-2.44) | 1.57 (1.21-2.03) | 0.16 |  |  |  |
|  |  | Model 2^1,3^ | | 2.11 (1.59-2.81) | 1.95 (1.50-2.54) | 1.70 (1.31-2.22) | 1.48 (1.09-2.01) | 0.23 |  |  |  |
|  | OR for CRP ≥3 mg/L^3^ | | | 1.00 | 0.91 (0.41-2.00) | 0.70 (0.30-1.61) | 0.66 (0.28-1.60) | 0.29 |  | 0.89 (0.65-1.20) | 0.43 |
| Homovanillic acid | | | |  |  |  |  |  |  |  |  |
|  | n | | | 80 | 85 | 71 | 78 |  |  |  |  |
|  | CRP, mg/L | | |  |  |  |  |  |  |  |  |
|  |  | Geometric mean | | 1.77 | 1.94 | 2.23 | 2.11 | - |  |  |  |
|  |  | Model 1^1,2^ | | 1.77 (1.40-2.26) | 1.90 (1.51-2.40) | 2.11 (1.63-2.73) | 1.89 (1.48-2.42) | 0.78 |  |  |  |
|  |  | Model 2^1,3^ | | 1.78 (1.38-2.31) | 1.76 (1.36-2.28) | 1.85 (1.40-2.43) | 1.63 (1.23-2.17) | 0.91 |  |  |  |
|  | OR for CRP ≥3 mg/L^3^ | | | 1.00 | 0.66 (0.30-1.45) | 1.22 (0.55-2.71) | 0.98 (0.43-2.28) | 0.71 |  | 1.02 (0.75-1.40) | 0.90 |
| 3,4-Dihydroxyphenylpropionic acid | | | | |  |  |  |  |  |  |  |
|  | n | | | 72 | 72 | 70 | 72 |  |  |  |  |
|  | CRP, mg/L | | |  |  |  |  |  |  |  |  |
|  |  | Geometric mean | | 2.69 | 1.79 | 1.99 | 1.79 | - |  |  |  |
|  |  | Model 1^1,2^ | | 2.81 (2.19-3.61) | 1.63 (1.27-2.08) | 1.77 (1.38-2.29) | 1.62 (1.25-2.09) | **0.004** |  |  |  |
|  |  | Model 2^1,3^ | | 2.60 (1.98-3.41) | 1.48 (1.14-1.93) | 1.63 (1.23-2.17) | 1.46 (1.10-1.93) | **0.003** |  |  |  |
|  | OR for CRP ≥3 mg/L^3^ | | | 1.00 | 0.57 (0.25-1.30) | 0.24 (0.10-0.57) | 0.27 (0.11-0.66) | **<0.001** |  | 0.63 (0.46-0.87) | **0.005** |
| 3,5-Dihydroxyphenylpropionic acid | | | | |  |  |  |  |  |  |  |
|  | n | | | 78 | 80 | 78 | 79 |  |  |  |  |
|  | CRP, mg/L | | |  |  |  |  |  |  |  |  |
|  |  | Geometric mean | | 1.96 | 2.34 | 1.95 | 1.78 | - |  |  |  |
|  |  | Model 1^1,2^ | | 2.15 (1.64-2.83) | 2.14 (1.70-2.71) | 1.72 (1.34-2.21) | 1.62 (1.25-2.09) | 0.30 |  |  |  |
|  |  | Model 2^1,3^ | | 2.04 (1.53-2.73) | 1.92 (1.49-2.48) | 1.58 (1.21-2.06) | 1.44 (1.06-1.95) | 0.26 |  |  |  |
|  | OR for CRP ≥3 mg/L^3^ | | | 1.00 | 0.81 (0.36-1.83) | 0.40 (0.16-1.00) | 0.31 (0.11-0.86) | **0.010** |  | 0.58 (0.39-0.86) | **0.007** |
| p-Coumaric acid | | | |  |  |  |  |  |  |  |  |
|  | n | | | 79 | 77 | 78 | 78 |  |  |  |  |
|  | CRP, mg/L | | |  |  |  |  |  |  |  |  |
|  |  | Geometric mean | | 2 | 2.43 | 1.71 | 1.9 | - |  |  |  |
|  |  | Model 1^1,2^ | | 2.00 (1.58-2.53) | 2.32 (1.82-2.97) | 1.55 (1.21-1.99) | 1.65 (1.27-2.15) | 0.07 |  |  |  |
|  |  | Model 2^1,3^ | | 1.85 (1.43-2.40) | 1.98 (1.52-2.59) | 1.43 (1.08-1.87) | 1.59 (1.19-2.13) | 0.22 |  |  |  |
|  | OR for CRP ≥3 mg/L^3^ | | | 1.00 | 1.08 (0.49-2.37) | 0.57 (0.24-1.37) | 0.95 (0.40-2.29) | 0.63 |  | 0.90 (0.65-1.25) | 0.53 |
| m-Coumaric acid | | | |  |  |  |  |  |  |  |  |
|  | n | | | 79 | 79 | 74 | 78 |  |  |  |  |
|  | CRP, mg/L | | |  |  |  |  |  |  |  |  |
|  |  | Geometric mean | | 2.07 | 1.91 | 1.97 | 1.98 | - |  |  |  |
|  |  | Model 1^1,2^ | | 1.98 (1.57-2.49) | 1.78 (1.40-2.25) | 1.87 (1.45-2.41) | 1.82 (1.41-2.35) | 0.92 |  |  |  |
|  |  | Model 2^1,3^ | | 1.76 (1.37-2.27) | 1.63 (1.25-2.13) | 1.76 (1.34-2.32) | 1.67 (1.26-2.21) | 0.95 |  |  |  |
|  | OR for CRP ≥3 mg/L^3^ | | | 1.00 | 1.07 (0.49-2.34) | 1.44 (0.66-3.15) | 1.21 (0.54-2.72) | 0.50 |  | 1.00 (0.75-1.34) | 0.98 |
| Caffeic acid | | | |  |  |  |  |  |  |  |  |
|  | n | | | 78 | 81 | 78 | 78 |  |  |  |  |
|  | CRP, mg/L | | |  |  |  |  |  |  |  |  |
|  |  | Geometric mean | | 2.04 | 2.18 | 1.87 | 1.91 | - |  |  |  |
|  |  | Model 1^1,2^ | | 2.01 (1.57-2.58) | 2.08 (1.65-2.64) | 1.86 (1.46-2.37) | 1.68 (1.31-2.16) | 0.61 |  |  |  |
|  |  | Model 2^1,3^ | | 2.03 (1.55-2.66) | 1.93 (1.48-2.51) | 1.61 (1.22-2.11) | 1.54 (1.18-2.00) | 0.30 |  |  |  |
|  | OR for CRP ≥3 mg/L^3^ | | | 1.00 | 0.90 (0.42-1.93) | 0.45 (0.20-1.02) | 0.37 (0.16-0.87) | **0.007** |  | 0.69 (0.51-0.93) | **0.015** |
| Ferulic acid | | | |  |  |  |  |  |  |  |  |
|  | n | | | 80 | 76 | 78 | 79 |  |  |  |  |
|  | CRP, mg/L | | |  |  |  |  |  |  |  |  |
|  |  | Geometric mean | | 1.84 | 2.24 | 1.94 | 2.01 | - |  |  |  |
|  |  | Model 1^1,2^ | | 1.90 (1.46-2.46) | 2.29 (1.79-2.93) | 1.69 (1.30-2.20) | 1.68 (1.28-2.21) | 0.30 |  |  |  |
|  |  | Model 2^1,3^ | | 1.84 (1.38-2.44) | 2.18 (1.66-2.88) | 1.55 (1.18-2.04) | 1.47 (1.10-1.96) | 0.16 |  |  |  |
|  | OR for CRP ≥3 mg/L^3^ | | | 1.00 | 1.12 (0.51-2.46) | 0.66 (0.25-1.72) | 0.44 (0.16-1.23) | 0.10 |  | 0.65 (0.44-0.96) | **0.030** |
| **Lignans** | | | |  |  |  |  |  |  |  |  |
| Enterodiol | | | |  |  |  |  |  |  |  |  |
|  | n | | | 82 | 71 | 73 | 76 |  |  |  |  |
|  | CRP, mg/L | | |  |  |  |  |  |  |  |  |
|  |  | Geometric mean | | 1.94 | 2.24 | 1.93 | 1.77 | - |  |  |  |
|  |  | Model 1^1,2^ | | 1.90 (1.50-2.41) | 2.07 (1.61-2.65) | 1.78 (1.39-2.28) | 1.76 (1.37-2.27) | 0.77 |  |  |  |
|  |  | Model 2^1,3^ | | 1.87 (1.44-2.41) | 1.89 (1.44-2.47) | 1.58 (1.21-2.07) | 1.62 (1.21-2.17) | 0.64 |  |  |  |
|  | OR for CRP ≥3 mg/L^3^ | | | 1.00 | 0.63 (0.29-1.40) | 0.36 (0.15-0.84) | 0.55 (0.23-1.29) | 0.077 |  | 0.77 (0.56-1.05) | 0.100 |
| Enterolactone | | | |  |  |  |  |  |  |  |  |
|  | n | | | 78 | 80 | 78 | 79 |  |  |  |  |
|  | CRP, mg/L | | |  |  |  |  |  |  |  |  |
|  |  | Geometric mean | | 2.41 | 2.11 | 1.89 | 1.66 | - |  |  |  |
|  |  | Model 1^1,2^ | | 2.61 (2.02-3.37) | 2.02 (1.60-2.55) | 1.81 (1.44-2.28) | 1.46 (1.15-1.86) | **0.010** |  |  |  |
|  |  | Model 2^1,3^ | | 2.17 (1.65-2.85) | 1.98 (1.52-2.57) | 1.60 (1.24-2.07) | 1.43 (1.09-1.87) | 0.068 |  |  |  |
|  | OR for CRP ≥3 mg/L^3^ | | | 1.00 | 1.05 (0.48-2.27) | 0.36 (0.15-0.83) | 0.46 (0.19-1.10) | **0.014** |  | 0.75 (0.55-1.02) | 0.069 |
| **Tyrosols** | | | |  |  |  |  |  |  |  |  |
| Tyrosol | | | |  |  |  |  |  |  |  |  |
|  | n | | | 86 | 74 | 79 | 74 |  |  |  |  |
|  | CRP, mg/L | | |  |  |  |  |  |  |  |  |
|  |  | Geometric mean | | 2.12 | 2.2 | 2.05 | 1.66 | - |  |  |  |
|  |  | Model 1^1,2^ | | 2.01 (1.61-2.51) | 2.04 (1.56-2.67) | 1.93 (1.51-2.47) | 1.56 (1.21-2.02) | 0.40 |  |  |  |
|  |  | Model 2^1,3^ | | 1.73 (1.34-2.23) | 1.84 (1.38-2.47) | 1.93 (1.48-2.50) | 1.48 (1.13-1.95) | 0.44 |  |  |  |
|  | OR for CRP ≥3 mg/L^3^ | | | 1.00 | 1.17 (0.53-2.57) | 1.31 (0.61-2.85) | 0.53 (0.22-1.25) | 0.27 |  | 0.86 (0.64-1.17) | 0.33 |
| Hydroxytyrosol | | | |  |  |  |  |  |  |  |  |
|  | n | | | 68 | 70 | 66 | 68 |  |  |  |  |
|  | CRP, mg/L | | |  |  |  |  |  |  |  |  |
|  |  | Geometric mean | | 2.35 | 2.3 | 2.02 | 1.69 | - |  |  |  |
|  |  | Model 1^1,2^ | | 2.31 (1.75-3.04) | 2.18 (1.68-2.84) | 1.86 (1.43-2.42) | 1.64 (1.27-2.13) | 0.25 |  |  |  |
|  |  | Model 2^1,3^ | | 1.88 (1.40-2.52) | 2.06 (1.54-2.75) | 1.71 (1.28-2.29) | 1.57 (1.19-2.07) | 0.47 |  |  |  |
|  | OR for CRP ≥3 mg/L^3^ | | | 1.00 | 0.73 (0.32-1.68) | 0.53 (0.22-1.28) | 0.34 (0.14-0.85) | **0.017** |  | 0.67 (0.48-0.93) | **0.016** |

^1^ values are geometric means (95% Confidence Interval), n varies in between polyphenols
^2^ adjusted for age, sex, country, and total energy intake
^3^ adjusted for age, sex, country, total energy intake, diabetes, cardiovascular problems, education, smoking status, alcohol intake, red and processed meat consumption, total fibre consumption, fish and shellfish intake, total physical activity, and BMI-adjusted waist circumference

^4^Standard deviations of individual polyphenols were as follows: Kaempferol (32.8); Quercetin (136.8); Catechin (21.3); Epicatechin (43.5); Apigenin (2.9); Naringenin (38.8); Hesperetin (45.2); Daidzein (72.4); Genistein (38.3); Equol (2.8); 4-Hydroxibenzoic acid (86.4); 3-Hydroxibenzoic acid (19.0); Protocatechuic acid (23.5); Gallic acid (29.5); Vanillic acid (121.0); 3,5-Dihydroxibenzoic acid (54.8); 4-Hydroxiphenylacetic acid (179.8); 3-Hydroxiphenylacetic acid (80.4); 3,4-Dihydroxiphenylacetic acid (18.4); Homovanillic acid (45.9); 3,4-Dihydroxiphenylpropionic acid (76.3); 3,5-Dihydroxiphenylpropionic acid (50.4); p-Coumaric acid (12.2); m-Coumaric acid (21.9); Caffeic acid (92.0); Ferulic acid (137.5); Enterodiol acid (6.8); Enterolactone acid (21.2); Resveratrol (6.0); Tyrosol (6.2); Hydroxityrosol (20.6)
