# Supplementary material for: Plasma polyphenols associated with lower high-sensitivity C-reactive protein concentrations: a cross-sectional study within the European Prospective Investigation into Cancer and Nutrition (EPIC) cohort
Source: Br J Nutr. 2020 Jan 28;123(2):198–208. doi: 10.1017/S0007114519002538 (PMC7015881; doi:10.1017/S0007114519002538)
Supplement: Supplementary file 1 [file S0007114519002538sup001.zip › S0007114519002538supp001.pptx]

## Slide 1
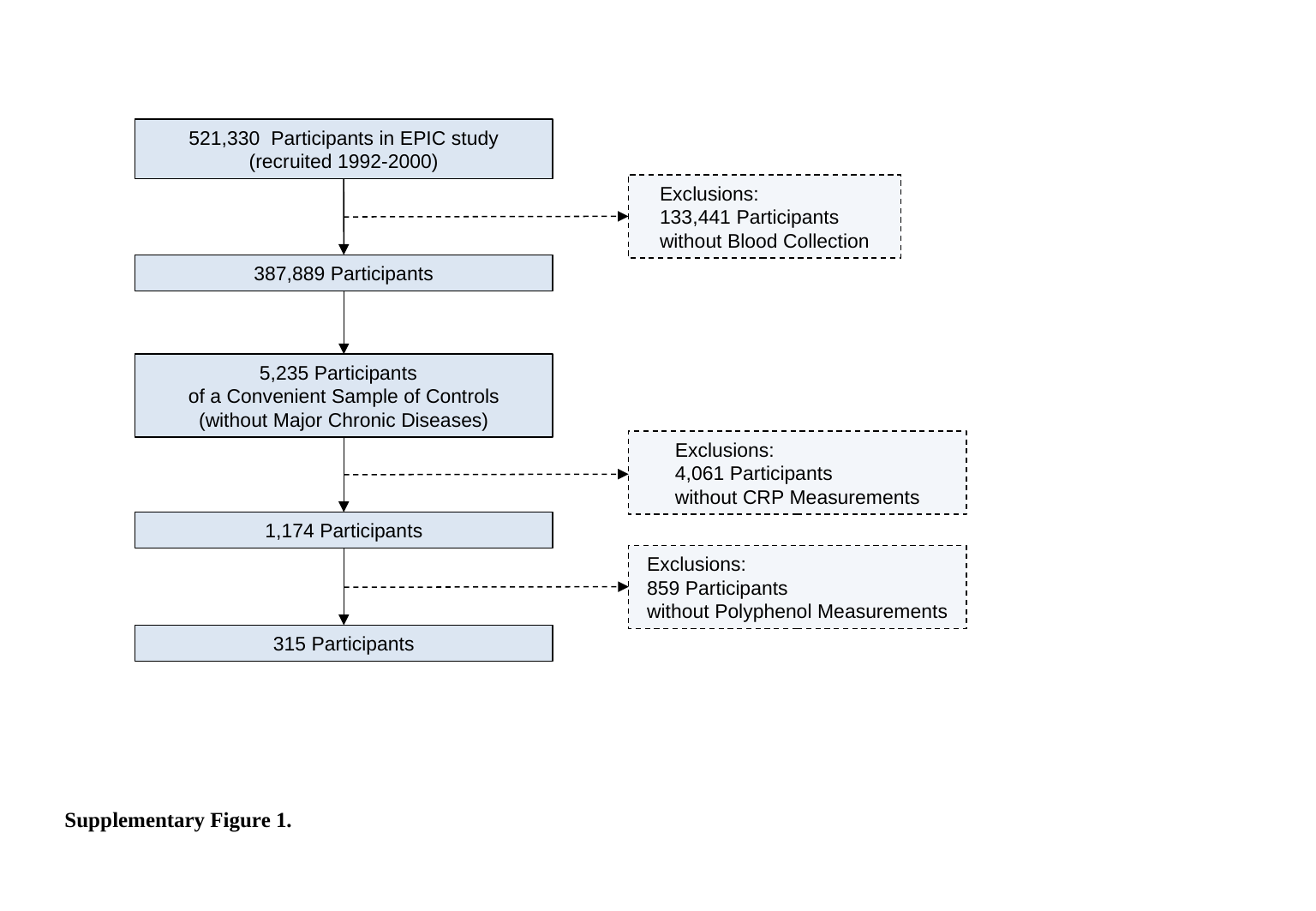

521,330 Participants in EPIC study
(recruited 1992-2000)
Exclusions:
133,441 Participants
without Blood Collection
387,889 Participants
5,235 Participants
of a Convenient Sample of Controls
(without Major Chronic Diseases)
Exclusions:
4,061 Participants
without CRP Measurements
1,174 Participants
Exclusions:
859 Participants
without Polyphenol Measurements
315 Participants
Supplementary Figure 1.
