# Supplementary material for: Plasma polyphenols associated with lower high-sensitivity C-reactive protein concentrations: a cross-sectional study within the European Prospective Investigation into Cancer and Nutrition (EPIC) cohort
Source: Br J Nutr. 2020 Jan 28;123(2):198–208. doi: 10.1017/S0007114519002538 (PMC7015881; doi:10.1017/S0007114519002538)
Supplement: Supplementary file 1 [file S0007114519002538sup001.zip › S0007114519002538supp002.pptx]

## Slide 1
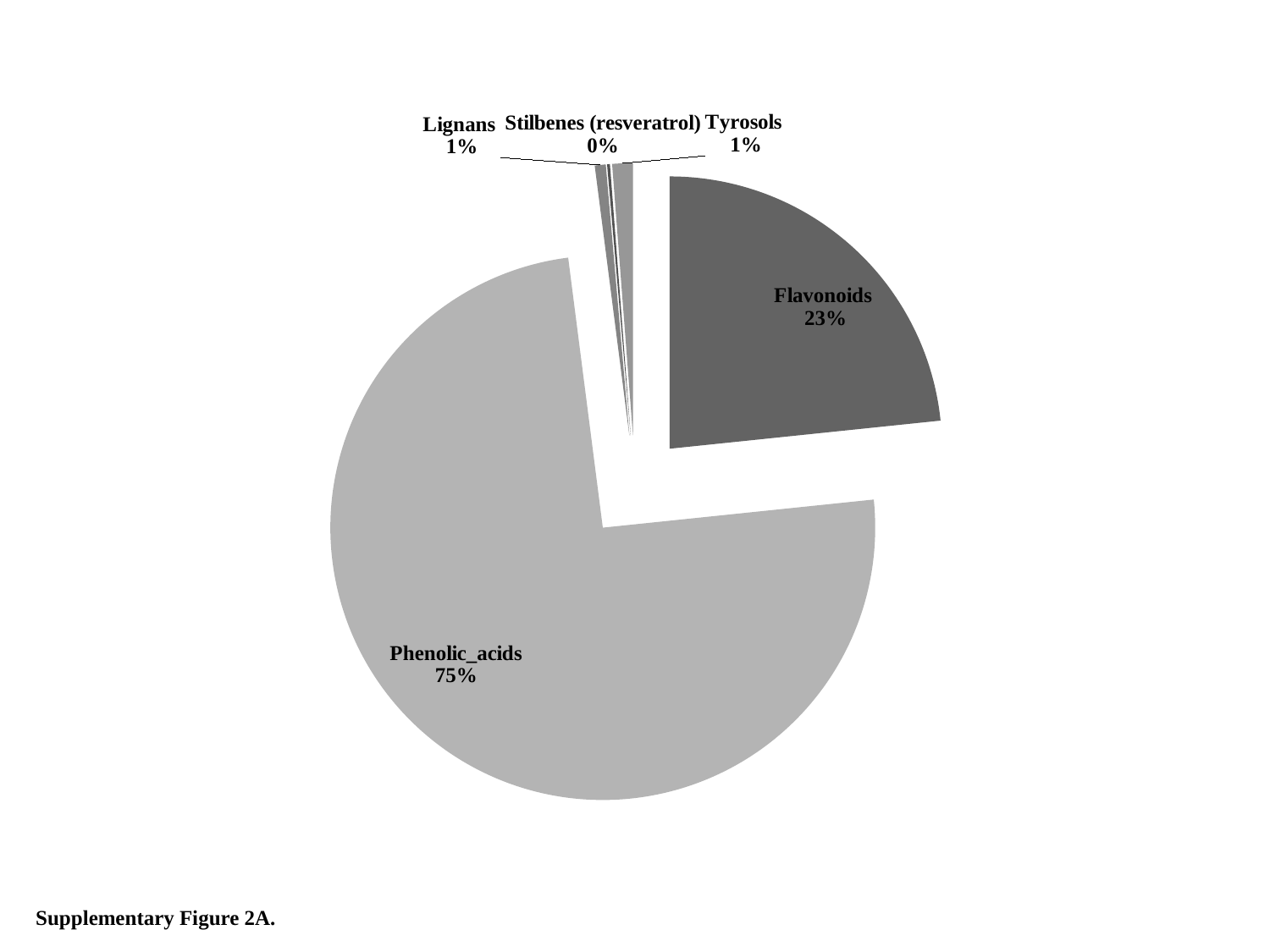

### Chart
| Category | Mean proportion of total polyphenols [%] |
|---|---|
| Flavonoids | 24.14 |
| Phenolic_acids | 77.13 |
| Lignans | 0.67 |
| Stilbenes (resveratrol) | 0.17 |
| Tyrosols | 1.26 |Supplementary Figure 2A.

## Slide 2
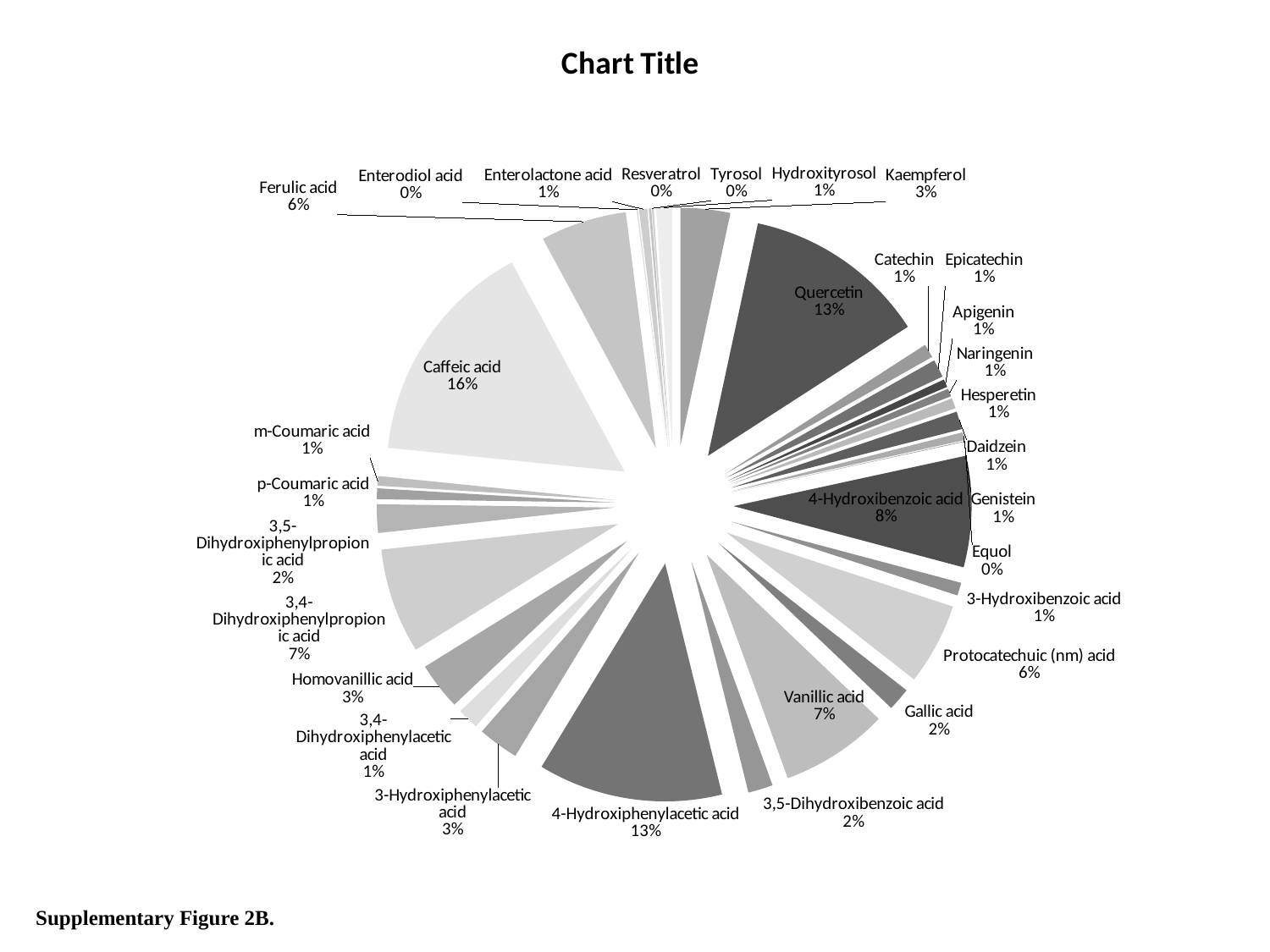

### Chart:
| Category | |
|---|---|
| | None |
| | None |
| | None |
| | None |
| | None |
| Kaempferol | 3.49 |
| Quercetin | 12.95 |
| Catechin | 0.97 |
| Epicatechin | 1.26 |
| Apigenin | 0.56 |
| Naringenin | 0.56 |
| Hesperetin | 0.73 |
| Daidzein | 1.26 |
| Genistein | 0.56 |
| Equol | 0.03 |
| 4-Hydroxibenzoic acid | 7.82 |
| 3-Hydroxibenzoic acid | 0.91 |
| Protocatechuic (nm) acid | 5.75 |
| Gallic acid | 1.62 |
| Vanillic acid | 7.59 |
| 3,5-Dihydroxibenzoic acid | 1.75 |
| 4-Hydroxiphenylacetic acid | 12.99 |
| 3-Hydroxiphenylacetic acid | 2.88 |
| 3,4-Dihydroxiphenylacetic acid | 1.51 |
| Homovanillic acid | 3.33 |
| 3,4-Dihydroxiphenylpropionic acid | 7.4 |
| 3,5-Dihydroxiphenylpropionic acid | 2.0 |
| p-Coumaric acid | 0.76 |
| m-Coumaric acid | 0.68 |
| Caffeic acid | 16.07 |
| Ferulic acid | 6.06 |
| Enterodiol acid | 0.09 |
| Enterolactone acid | 0.58 |
| Resveratrol | 0.17 |
| Tyrosol | 0.14 |
| Hydroxityrosol | 1.12 |Supplementary Figure 2B.
