# Supplementary material for: Plasma polyphenols associated with lower high-sensitivity C-reactive protein concentrations: a cross-sectional study within the European Prospective Investigation into Cancer and Nutrition (EPIC) cohort
Source: Br J Nutr. 2020 Jan 28;123(2):198–208. doi: 10.1017/S0007114519002538 (PMC7015881; doi:10.1017/S0007114519002538)
Supplement: Supplementary file 1 [file S0007114519002538sup001.zip › S0007114519002538supp003.pptx]

## Slide 1
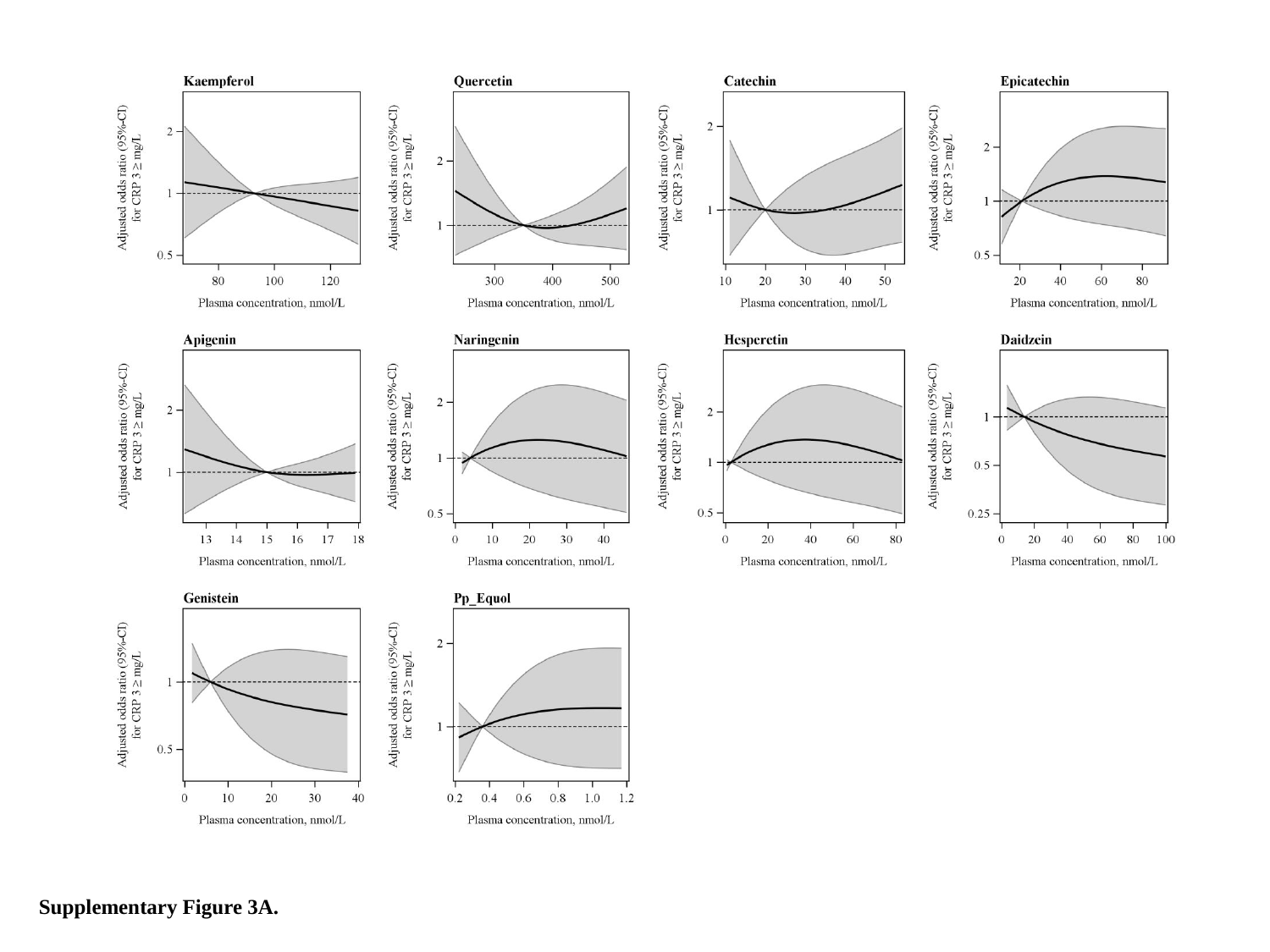

Supplementary Figure 3A.

## Slide 2
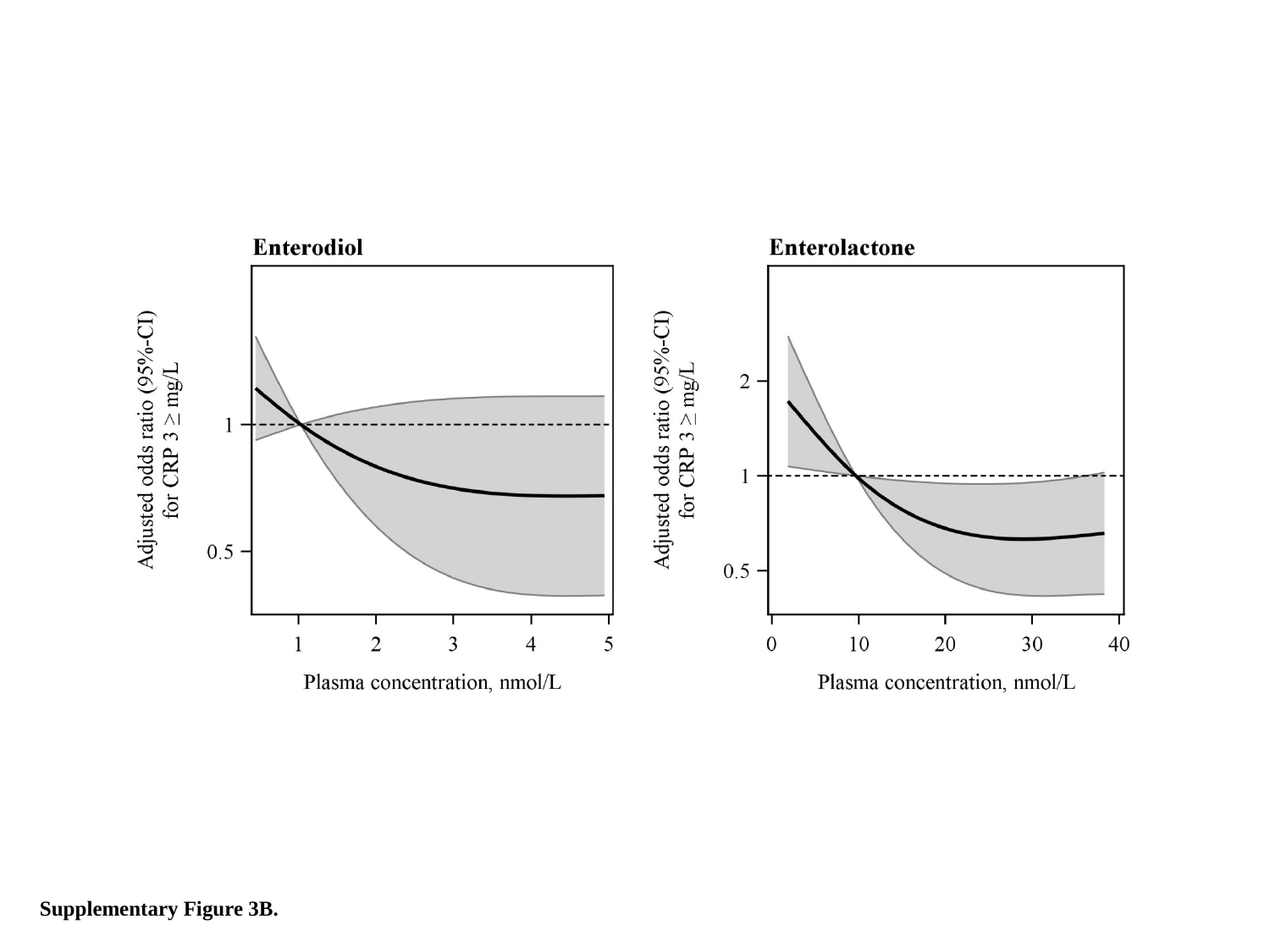

Supplementary Figure 3B.

## Slide 3
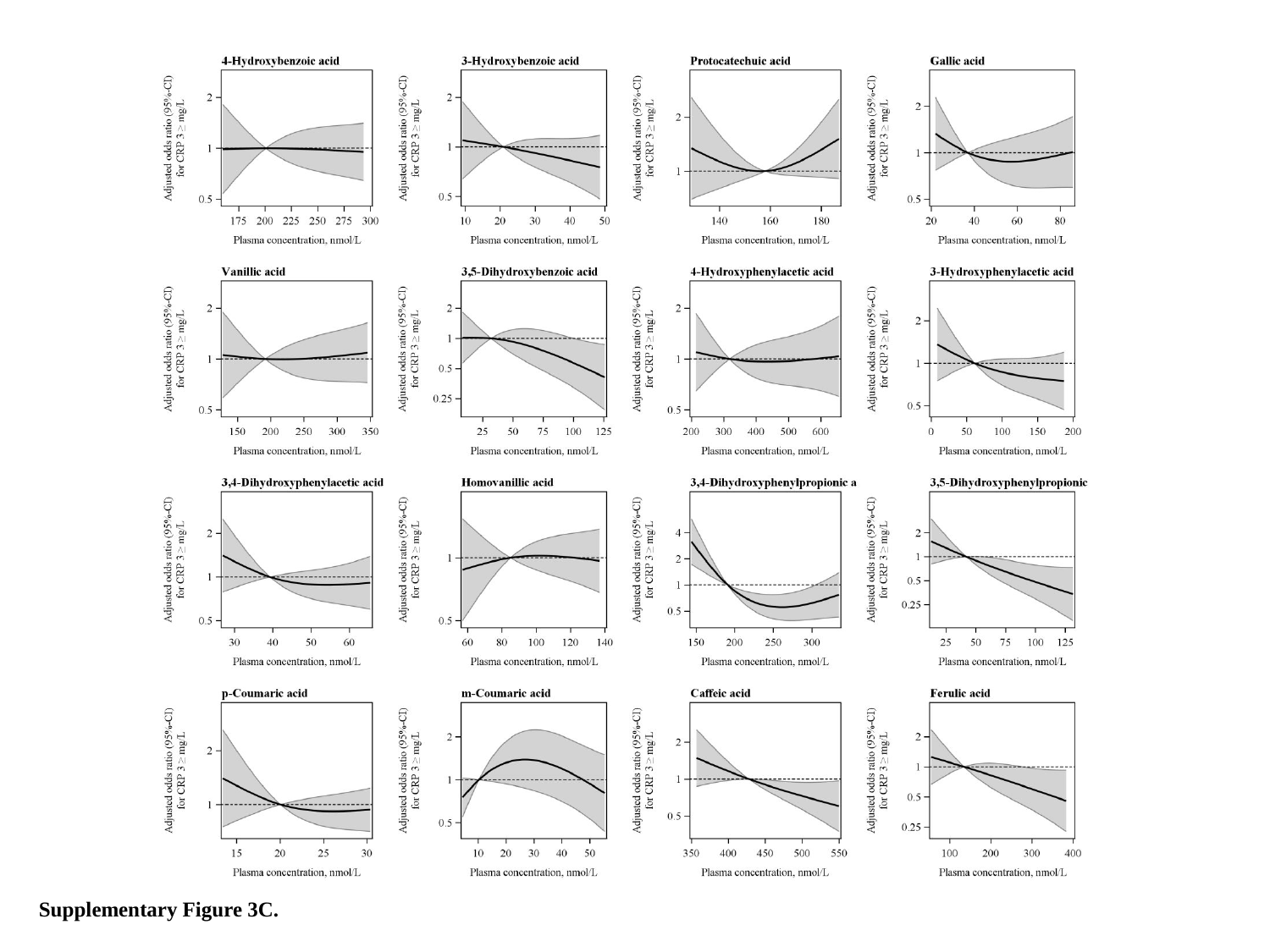

Supplementary Figure 3C.

## Slide 4
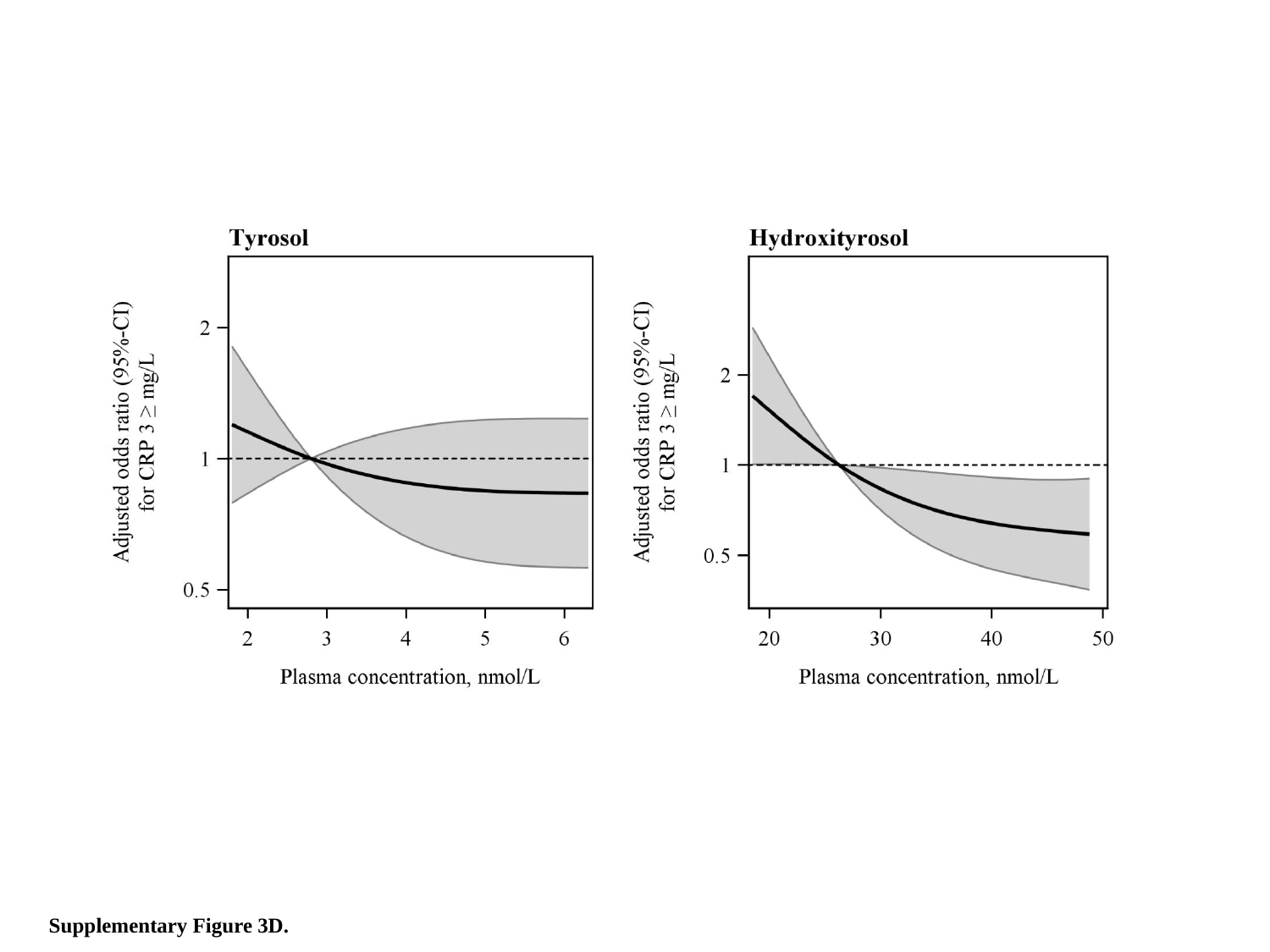

Supplementary Figure 3D.
